# Tumor-targeted therapy with BRAF-inhibitor recruits activated dendritic cells to promote tumor immunity in melanoma

**DOI:** 10.1136/jitc-2023-008606

**Published:** 2024-04-17

**Authors:** Florian Hornsteiner, Janine Vierthaler, Helen Strandt, Antonia Resag, Zhe Fu, Markus Ausserhofer, Christoph H Tripp, Sophie Dieckmann, Markus Kanduth, Kathryn Farrand, Sarah Bregar, Niloofar Nemati, Natascha Hermann-Kleiter, Athanasios Seretis, Sudhir Morla, David Mullins, Francesca Finotello, Zlatko Trajanoski, Guido Wollmann, Franca Ronchese, Marc Schmitz, Ian F Hermans, Patrizia Stoitzner

**Affiliations:** 1 Department of Dermatology, Venereology and Allergology, Medical University of Innsbruck, Innsbruck, Austria; 2 Institute of Immunology, Faculty of Medicine Carl Gustav Carus, Dresden University of Technology, Dresden, Germany; 3 Malaghan Institute of Medical Research, Wellington, New Zealand; 4 Department of Molecular Biology, Digital Science Center (DiSC), University of Innsbruck, Innsbruck, Austria; 5 Biocenter, Institute of Bioinformatics, Medical University of Innsbruck, Innsbruck, Austria; 6 Institute of Cell Genetics, Department for Genetics and Pharmacology, Medical University of Innsbruck, Innsbruck, Austria; 7 Institute for Biomedical Aging Research, University of Innsbruck, Innsbruck, Austria; 8 Institute of Virology, Medical University of Innsbruck, Innsbruck, Austria; 9 Department of Microbiology and Immunology, Geisel School of Medicine at Dartmouth, Hanover, New Hampshire, USA

**Keywords:** Skin Cancer, Dendritic cells, Myeloid cells, Immune modulatory, Tumor immunity, Tumor-targeted therapy

## Abstract

**Background:**

Tumor-targeted therapy causes impressive tumor regression, but the emergence of resistance limits long-term survival benefits in patients. Little information is available on the role of the myeloid cell network, especially dendritic cells (DC) during tumor-targeted therapy.

**Methods:**

Here, we investigated therapy-mediated immunological alterations in the tumor microenvironment (TME) and tumor-draining lymph nodes (LN) in the D4M.3A preclinical melanoma mouse model (harboring the V-Raf murine sarcoma viral oncogene homolog B (BRAF)^V600E^ mutation) by using high-dimensional multicolor flow cytometry in combination with multiplex immunohistochemistry. This was complemented with RNA sequencing and cytokine quantification to characterize the immune status of the tumors. The importance of T cells during tumor-targeted therapy was investigated by depleting CD4^+^ or CD8^+^ T cells in tumor-bearing mice. Tumor antigen-specific T-cell responses were characterized by performing in vivo T-cell proliferation assays and the contribution of conventional type 1 DC (cDC1) to T-cell immunity during tumor-targeted therapy was assessed using Batf3^−/−^ mice lacking cDC1.

**Results:**

Our findings reveal that BRAF-inhibitor therapy increased tumor immunogenicity, reflected by an upregulation of genes associated with immune activation. The T cell-inflamed TME contained higher numbers of activated cDC1 and cDC2 but also inflammatory CCR2-expressing monocytes. At the same time, tumor-targeted therapy enhanced the frequency of migratory, activated DC subsets in tumor-draining LN. Even more, we identified a cDC2 population expressing the Fc gamma receptor I (FcγRI)/CD64 in tumors and LN that displayed high levels of CD40 and CCR7 indicating involvement in T cell-mediated tumor immunity. The importance of cDC2 is underlined by just a partial loss of therapy response in a cDC1-deficient mouse model. Both CD4^+^ and CD8^+^ T cells were essential for therapy response as their respective depletion impaired therapy success. On resistance development, the tumors reverted to an immunologically inert state with a loss of DC and inflammatory monocytes together with the accumulation of regulatory T cells. Moreover, tumor antigen-specific CD8^+^ T cells were compromised in proliferation and interferon-γ-production.

**Conclusion:**

Our results give novel insights into the remodeling of the myeloid landscape by tumor-targeted therapy. We demonstrate that the transient immunogenic tumor milieu contains more activated DC. This knowledge has important implications for the development of future combinatorial therapies.

**WHAT IS ALREADY KNOWN ON THIS TOPIC**

- Several studies demonstrated in patients with melanoma and mouse models that V-Raf murine sarcoma viral oncogene homolog B (BRAF)-inhibitor therapy increased the expression of tumor antigens and recruitment of T cells, while the numbers of regulatory T cells and myeloid-derived suppressor cells decreased.

**WHAT THIS STUDY ADDS**

- In our study, we now add novel insights on the profound remodeling of myeloid subtypes in a transplantable melanoma mouse model characterized by the recruitment of activated, inflammatory monocytes and dendritic cell (DC) subsets to the T cell-inflamed tumor tissue. We identified a highly activated, migratory conventional type 2 DC (cDC2) population expressing the Fc gamma receptor I (FcγRI)/CD64 in tumors and lymph nodes correlating with enhanced antitumor immunity in lymph nodes. During resistance development, tumors revert to an immunologically inert state with the loss of activated DC.

**HOW THIS STUDY MIGHT AFFECT RESEARCH, PRACTICE OR POLICY**

- Our findings have implications for the careful timing of tumor-targeted therapy with immunotherapy to benefit from the highly immunogenic milieu early on during treatment. The role of the cDC2 population in antitumor immunity needs more attention to harness their potential for future immunotherapies. Follow-up studies should explore the potential of DC-based immunotherapeutic approaches in combination with tumor-targeted therapy to improve tumor immunity and delay resistance development.

## Introduction

Melanoma comprises a small fraction of all cutaneous malignancies but is responsible for the majority of skin cancer-related deaths. Melanoma has a high mutational load with driver mutations occurring in genes regulating several crucial signaling pathways involved in proliferation, growth and metabolism such as in the mitogen-activated protein kinase (MAPK) pathway (eg, V-Raf murine sarcoma viral oncogene homolog B (*BRAF)*), and the phosphoinositide 3-kinase pathway (eg, Phosphatase and TENsin homolog deleted on chromosome 10 (*PTEN*)).^1^ Half of the patients with melanoma carry mutations affecting the *BRAF* gene, leading to an amino acid substitution of valine to glutamic acid in position 600 (BRAF^V600E^), resulting in a constitutive activation of the MAPK pathway.^2^ Great progress in the treatment of patients with BRAF-mutant melanoma has been achieved with the introduction of selective BRAF inhibitors (BRAFi).^3 4^ Although BRAFi induce impressive melanoma regression, therapy resistance develops within the first year of treatment caused by several mechanisms including Neuroblastoma RAS viral oncogene homolog (*NRAS)* mutations, aberrant *BRAF* splicing or *BRAF* amplification, resulting in the reactivation of the MAPK signal transduction cascade.^5 6^

Besides inducing programmed cell death of BRAF^V600E^ mutant melanoma cells, BRAF inhibition shapes the tumor microenvironment (TME), thereby influencing tumor immunogenicity.^7 8^ In patients with melanoma, tumor-targeted therapy increased the expression of tumor antigens and infiltration of activated T cells,^9–11^ while the number of myeloid-derived suppressor cells (MDSC) decreased.^12^ Likewise, BRAFi-treated transplantable and autochthonous melanoma mouse models showed recruitment of activated T and natural killer (NK) cells, whereas regulatory T cells (Treg) and MDSC were decreased.^13–17^

The most abundant immune cells in the TME are a complex mix of myeloid subtypes, their definition in tumors is rather inconsistent.^18–20^ Monocytes, neutrophils, MDSC and tumor-associated macrophages (TAM) are often characterized by their immunosuppressive capacity on the antitumor immune response.^21^ Within the myeloid network, dendritic cells (DC) are recognized as the most potent antigen-presenting cells of the immune system. DC can be divided into plasmacytoid DC (pDC) and conventional DC (cDC), which can be further subdivided into cDC1 and cDC2. Their unique ability to present tumor antigens to induce T cell immunity renders them key regulators in the context of cancer.^22 23^ DC capture antigens from tumor cells within the TME and migrate to the tumor-draining lymph nodes (LN), where antigen presentation occurs.^24^ The cDC1 subset is the main producer of interleukin (IL)-12 and specialized in the cross-presentation of tumor antigens to CD8^+^ T cells demonstrated by multiple studies.^18 19 25–27^ Recently, their importance in the induction of CD4^+^ T cell immunity was reported^28^ arguing that in tumors cDC1 are needed for both CD4^+^ and CD8^+^ T cell responses. In contrast, cDC2 are responsible for polarizing CD4^+^ T helper cell responses^23^ and their role in unleashing antitumor CD4^+^ T-cell immunity was recently demonstrated.^29^ However, cDC2 can also present tumor antigens to CD8^+^ T cells^20 30^ underlining the versatility of DC subsets in T cell priming.

Despite their relevance in tumor immunity, the role of DC in tumor-targeted therapy is still poorly defined. In-depth characterization of alterations in the myeloid immune landscape during tumor-targeted therapy of melanoma is crucial for the advancement of future treatment combinations with immunotherapy. Therefore, we studied the alterations in the myeloid landscape in the TME of the transplantable D4M.3A preclinical mouse model of melanoma harboring the BRAF^V600E^ mutation and PTEN loss. Our findings reveal that BRAFi caused a transient inflamed tumor milieu recruiting activated monocytes, DC and effector T cells to the TME. Even more, the appearance of a highly activated cDC2 population expressing the Fc gamma receptor I (FcγRI)/CD64 in BRAFi-treated tumors underlines the complexity of the myeloid network in tumors. During resistance development to BRAFi the accumulation of intratumoral Treg and compromised tumor-specific CD8^+^ T-cell responses in tumor-draining LN argue for loss of treatment-induced tumor immunogenicity. Thus, our work adds novel insights to the complexity of immunological effects induced by tumor-targeted therapy by pointing at potential roles for DC and inflammatory myeloid subsets in therapy-induced tumor immunity.

## Results

### Tumor-targeted therapy with BRAFi creates an immunogenic TME in melanoma

**Figure 1:**
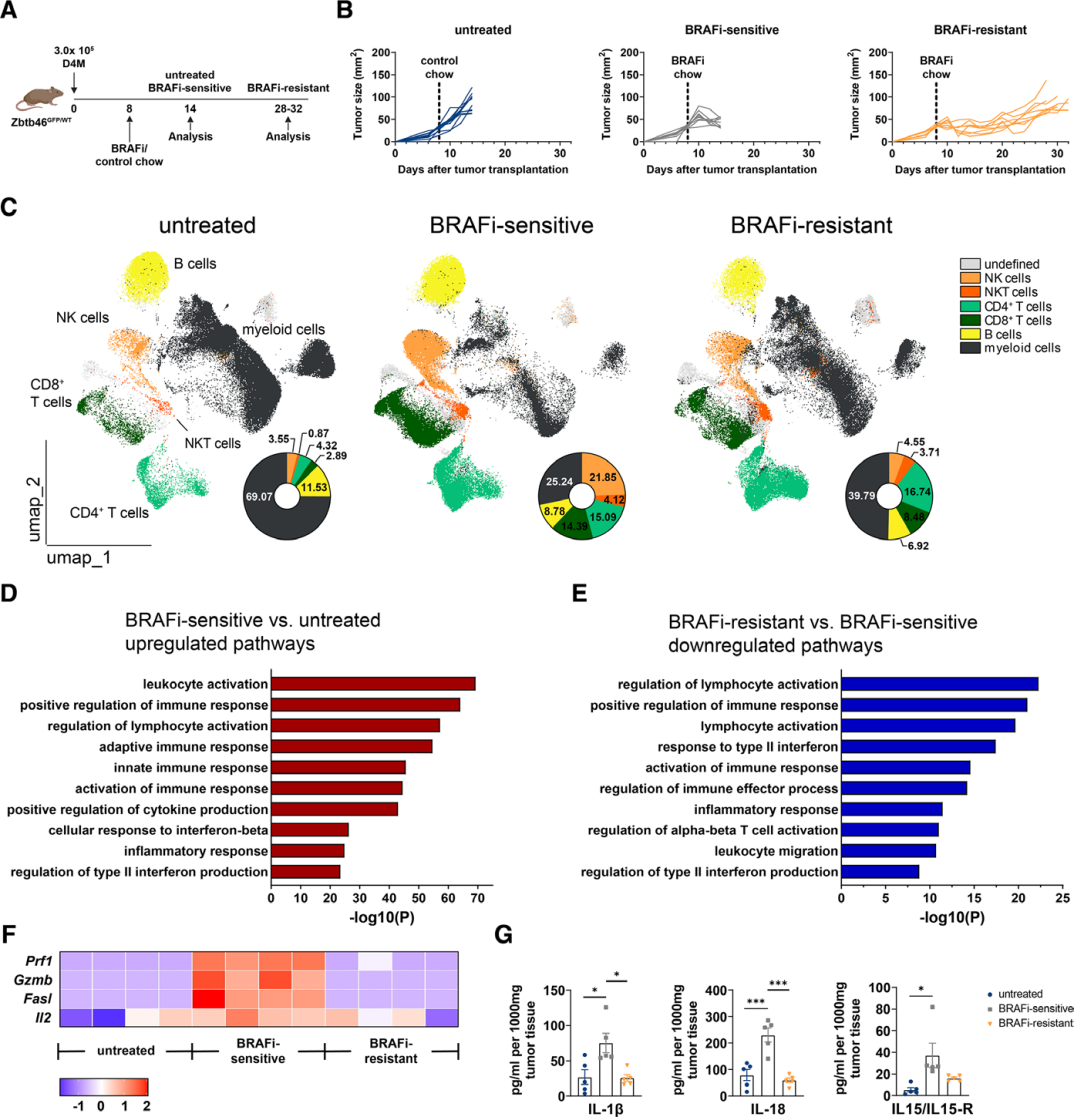
BRAFi treatment mediates the strong immunomodulatory activity. (A) Experimental design: 3×10^5^ D4M melanoma cells were subcutaneously injected into the flank skin of Zbtb46^GFP/WT^ mice. When tumors reached a size of 30–35 mm^2^ on day 8 after transplantation, mice received either BRAFi-containing or control chow. On day 14, untreated and BRAFi-sensitive D4M tumors were analyzed. Another group of mice kept on the BRAFi-containing diet started to regrow tumors due to BRAFi-resistance (analyzed on days 28–32 after transplantation when tumors were comparable in size to untreated ones, BRAFi-resistant). (B) Individual D4M tumor growth of three independent experiments is shown (n≥9/group). (C) Relative abundance of tumor-infiltrating NK cells, NKT cells, CD4^+^ T cells, CD8^+^ T cells, B cells and myeloid cells from untreated, BRAFi-sensitive and BRAFi-resistant tumors was measured by flow cytometry analysis. Shown is a UMAP dimensionality reduction of three representative mice per group. (D,E) Gene Set Enrichment Analysis was performed on bulk RNA sequencing data from untreated, BRAFi-sensitive and BRAFi-resistant tumor tissue. Barplots report the log-scaled p values of the most enriched terms in BRAFi-sensitive tumors compared with untreated tumors (D) and BRAFi-sensitive tumors compared with BRAFi-resistant tumors (E) based on differential expression analysis. (F) The heatmap depicts normalized and relative expression levels (z-score) for a selection of genes important for immune cell-related cytotoxicity. (G) Protein levels for the cytokines IL-1β, IL-18 and IL-15/IL-15R were measured in tumor lysates (n=5/group). For (D–F), results from four mice/group are shown. Statistical significance was determined using one-way analysis of variance followed by Tukey’s multiple comparison test or Kruskal-Wallis test followed by Dunn’s multiple comparison test. Graphs show the mean±SEM. *p<0.05; ***p<0.001. BRAFi, V-Raf murine sarcoma viral oncogene homolog B inhibitors; IL, interleukin; NK, natural killer; NKT, natural killer T; UMAP, Uniform Manifold Approximation and Projection.

For this study, we used the BRAF^V600E^-mutant melanoma cell line D4M.3A (from now on called D4M), generated from a Tyr::CreER;Braf^CA^;Pten^lox/lox^ transgenic melanoma mouse model which demonstrated sensitivity to BRAFi.^31^ The D4M melanoma cells were subcutaneously (s.c.) injected into the flank skin of Zbtb46^GFP/WT^ mice, a cDC-specific reporter mouse model.^32^ When transplanted tumors reached a size of about 30–35 mm^2^ (8 days after tumor transplantation), animals were given either control chow or BRAFi-containing chow and analyzed at different time points (figure 1A). Untreated mice receiving control chow reached maximum tumor size within 14 days after tumor cell injection. Treatment with BRAFi-containing chow reduced tumor size within 1 week by roughly 50%, and these were analyzed as BRAFi-sensitive tumors by day 14 after transplantation at the same time point as control tumors. Another group of mice with continued BRAFi therapy showed resistance development after 2–3 weeks when they were analyzed as BRAFi-resistant tumors (figure 1B). BRAFi therapy prolonged survival of mice (online supplemental figure 1a) and changed the tumor weight according to therapy response (online supplemental figure 1b). The classification of the three experimental groups reflects the course of biopsy sampling under tumor-targeted therapy in patients with BRAF-mutant melanoma.^33^

10.1136/jitc-2023-008606.supp1 Supplementary data

Previous reports suggested that tumor-targeted therapy induces caspase-3 activation^34^ which can subsequently lead to pyroptosis initiated by caspase-3-mediated gasdermin E (GSDME) cleavage in response to various apoptotic stimuli.^35 36^ Indeed, we could confirm that BRAFi treatment of D4M melanoma cells in vitro induced caspase-3 cleavage as detected by capillary-based immmunoblotting with the JESS system, resulting in the cleavage of GSDME. This BRAFi-mediated tumor cell death was linked to the release of the inflammatory molecules High-Mobility-Group-Protein B1 (HMGB1) and Heat Shock Protein 90 (HSP90) from D4M melanoma cells (online supplemental figure 1c). This data indicates that tumor-targeted therapy with BRAFi induces GSDME-mediated immunogenic cell death and is in line with a recent publication.^33^

We studied the immunological alterations in the TME during BRAFi therapy with an adapted 24-color flow cytometry panel^37^ that we had established earlier for mouse skin and lymphatic tissue.^38^ This panel allows a detailed analysis of the different myeloid cell subsets with the simultaneous identification of NK cells, Natural Killer T (NKT) cells, T cells and B cells. We first focused on the lymphoid cells by identifying the major populations by manual gating (for gating strategy see online supplemental figure 1d,e) and overlaying them on the Uniform Manifold Approximation and Projection (UMAP) plots shown in figure 1C. Within the CD45^+^ compartment, we observed significantly increased percentages of NK and NKT cells as well as CD4^+^ and CD8^+^ T cells in BRAFi-sensitive tumors. Interestingly, in the BRAFi-resistant tumors fewer NK cells and CD8^+^ T cells were present in tumors, whereas NKT and CD4^+^ T-cell abundances were unchanged. For the myeloid cell compartment, there were fewer myeloid cells within the CD45^+^ immune cells during the BRAFi-sensitive phase, with a repopulation in resistant tumors (figure 1C, online supplemental figure 1f).

For deeper insights into BRAFi-mediated immunomodulation, bulk RNA sequencing (RNA-seq) analysis of whole tumor tissue was performed. For an overall view of immunological differences between untreated, BRAFi-sensitive and BRAFi-resistant tumors, we performed Gene Set Enrichment Analysis (GSEA).^39^ GSEA showed an enrichment in genes reflecting activation of immune cells and inflammatory responses, for example, leukocyte activation, positive regulation of lymphocyte activation or positive regulation of cytokine production. This underlines the more immunogenic profile of BRAFi-sensitive tumors compared with untreated ones. Moreover, BRAFi-sensitive tumors were enriched in gene sets related to the cellular response to interferon-beta and regulation of type II interferon production (figure 1D). In contrast, BRAFi-resistant tumors displayed a downregulation of gene sets linked to lymphocyte activation, response to type II interferon or inflammatory response, indicating that on tumor relapse the TME reverted to an inert state (figure 1E).

In support of our findings, the analysis of genes important for T cell function associated with immune cell-related cytotoxicity, like *Prf1*, *Gzmb*, *Fasl* and activation of T cells, like *Il2* were upregulated during BRAFi-sensitive phase and downregulated in resistant tumors (figure 1F). To investigate the TME in more detail on a protein level, we performed cytokine analysis by Bio-Plex technology. The increased levels of several pro-inflammatory cytokines, including IL-1β, IL-18 and IL-15/IL-15R reflect the immunogenic milieu induced by BRAFi early on during therapy, however, this effect is just transient as these cytokines are decreased in resistant tumors (figure 1G).

Collectively, this data highlights that in addition to potent tumor-intrinsic activity, tumor-targeted therapy of melanoma with BRAFi induces a pro-inflammatory TME as reflected by the enrichment of genes important for the activation of the immune system and inflammatory responses, cytokine production and recruitment of effector NK and T cells. During resistance development the TME reverted to an inert state more comparable to untreated tumors.

### BRAFi treatment creates a T cell-inflamed tumor milieu

**Figure 2:**
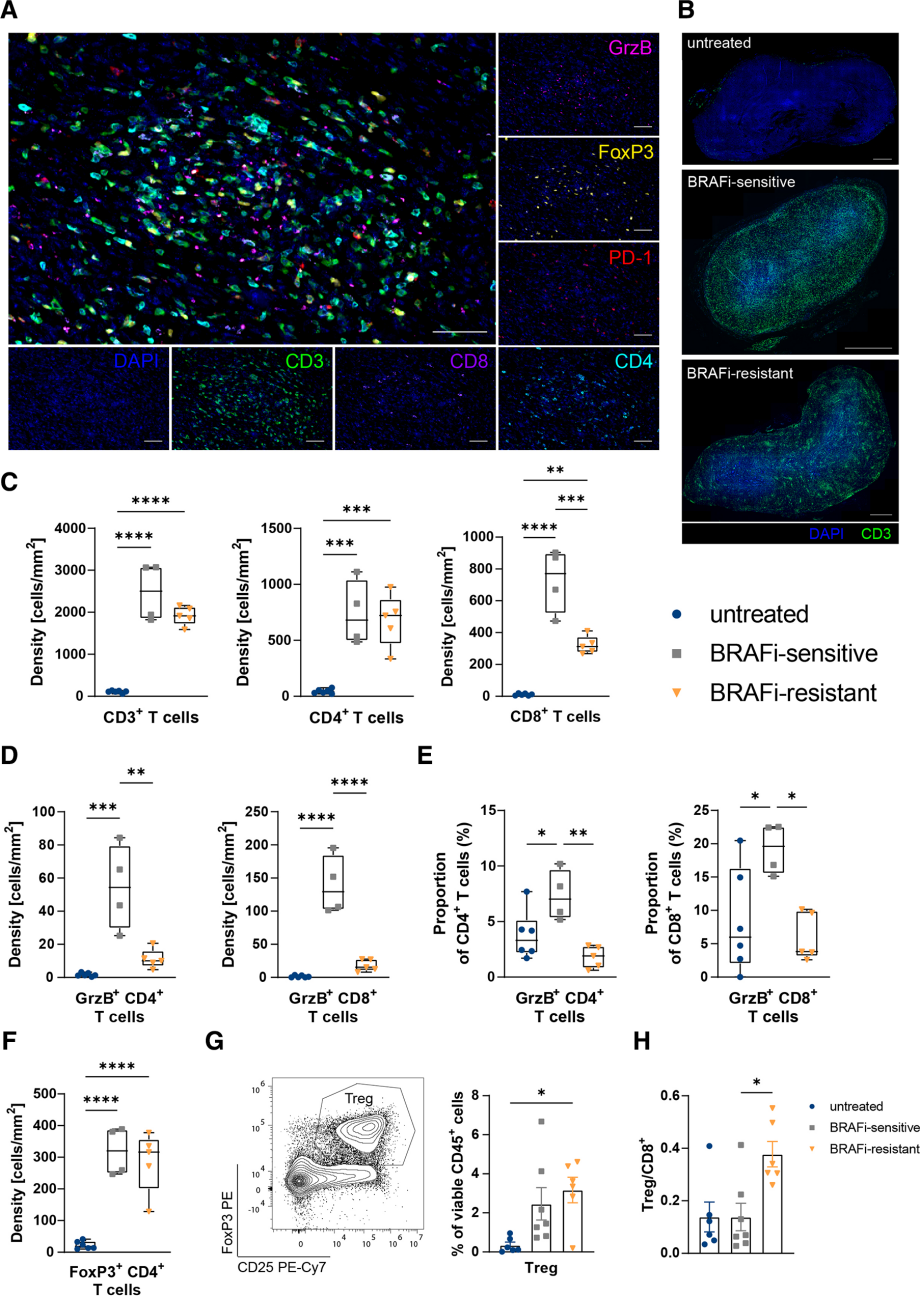
BRAFi treatment creates a T cell-inflamed tumor microenvironment. (A) Representative multicolor staining of tumor sections with a T cell panel including DAPI (blue), CD3 (green), CD8 (purple), CD4 (cyan), PD-1 (red), FoxP3 (yellow), and granzyme B (magenta). Scale bar indicates 50 µm. (B) Representative sections of untreated, BRAFi-sensitive and BRAFi-resistant tumors, demonstrating the localization of CD3^+^ T cells (green). Scale bar indicates 1 mm. (C) Densities (cells/mm^2^) were assessed for CD3^+^ T cells, CD4^+^ CD3^+^ T cells and CD8^+^ CD3^+^ T cells. (D) Densities (cells/mm^2^) of CD4^+^ and CD8^+^ T cells positive for GrzB. (E) Proportions of CD4^+^ and CD8^+^ T cells positive for GrzB. (F) Densities (cells/mm^2^) of CD4^+^ T cells positive for FoxP3. (G) Flow cytometry analysis to determine Treg in untreated, BRAFi-sensitive, and BRAFi-resistant tumors. (H) Flow cytometry analysis to calculate the ratio of Treg to CD8^+^ T cells. For (C–F) results from ≥4 mice/group are shown. For (G–H) summary graphs of two independent experiments are shown (n≥6/group). Statistical significance was determined using one-way analysis of variance followed by Tukey’s multiple comparison test or Kruskal-Wallis test followed by Dunn’s multiple comparison test. Graphs show the mean±SEM. *p<0.05; **p<0.01; ***p<0.001; ****p<0.0001. BRAFi, V-Raf murine sarcoma viral oncogene homolog B inhibitors; DAPI, 4′,6-Diamidin-2-phenylindol, DAPI; PD-1, Programmed Cell Death Protein-1; FoxP3, Forkhead-Box-Protein P3; GrzB, granzyme B; Treg, regulatory T cells.

Immune cells are often excluded from the tumor bed and cannot exert their function.^40^ To assess the localization, frequency and functional characteristics of T cells in BRAFi-treated D4M tumors, multiplex immunohistochemistry (mIHC) using a 6-marker panel was performed. For a comprehensive view of the tumor-infiltrating T cell landscape, the markers CD3, CD8, CD4, Programmed Cell Death Protein (PD)-1, granzyme B (GrzB) and Forkhead-Box-Protein P3 (FoxP3) were employed in addition to 4′,6-Diamidin-2-phenylindol (DAPI) nuclear staining (figure 2A, online supplemental figure 2a). Tumor-targeted therapy with BRAFi caused a switch from a T cell-excluded to a T cell-inflamed TME as we observed a redistribution of T cells from the tumor margin in untreated D4M tumors into the tumor center in BRAFi-sensitive tumors. Interestingly, the development of BRAFi-resistance relocated T cells in clusters (figure 2B). Consistent with flow cytometry analysis, CD4^+^ and CD8^+^ T cells were rare in untreated D4M tumors, but highly abundant in BRAFi-sensitive tumors (figure 2C). In regards to functional properties of infiltrating T cells, we observed a higher density and percentage of GrzB-positive CD4^+^ and CD8^+^ T cells during BRAFi-sensitive phase compared with untreated and BRAFi-resistant tumors (figure 2D,E). Furthermore, the frequency of PD-1 expressing CD4^+^ T cells and CD8^+^ T cells was higher during BRAFi treatment with a loss of PD-1^+^ CD8^+^ T cells in resistant tumors (online supplemental figure 2b). Proportions for PD-1 expressing CD4^+^ and CD8^+^ T cells were unchanged between the different treatment groups (online supplemental figure 2c).

Our flow cytometry data and microscopy stainings revealed that CD4^+^ T cells are still present in the resistant phase (figure 1C, online supplemental figure 1f, figure 2C). Therefore, we hypothesized that Treg infiltrate tumors during BRAFi treatment. Indeed, we detected significantly more Treg in BRAFi-sensitive tumor sections and they persisted when therapy resistance developed (figure 2F). Flow cytometry analysis confirmed the higher abundance of Treg on tumor-targeted therapy (figure 2G, online supplemental figure 2d), and furthermore, revealed the highest Treg/CD8^+^ T cell ratio in the resistant phase (figure 2H).

In summary, the immune modulation induced by BRAFi treatment creates a T cell-inflamed TME with many cytotoxic T cells entering the core of the tumors along an increasing frequency of Treg. This pattern changes during BRAFi resistance where Treg dominate over CD8^+^ T cells contributing to a potential immunosuppressive TME.

### BRAFi therapy transiently recruits inflammatory monocytes and pDC to the TME

**Figure 3:**
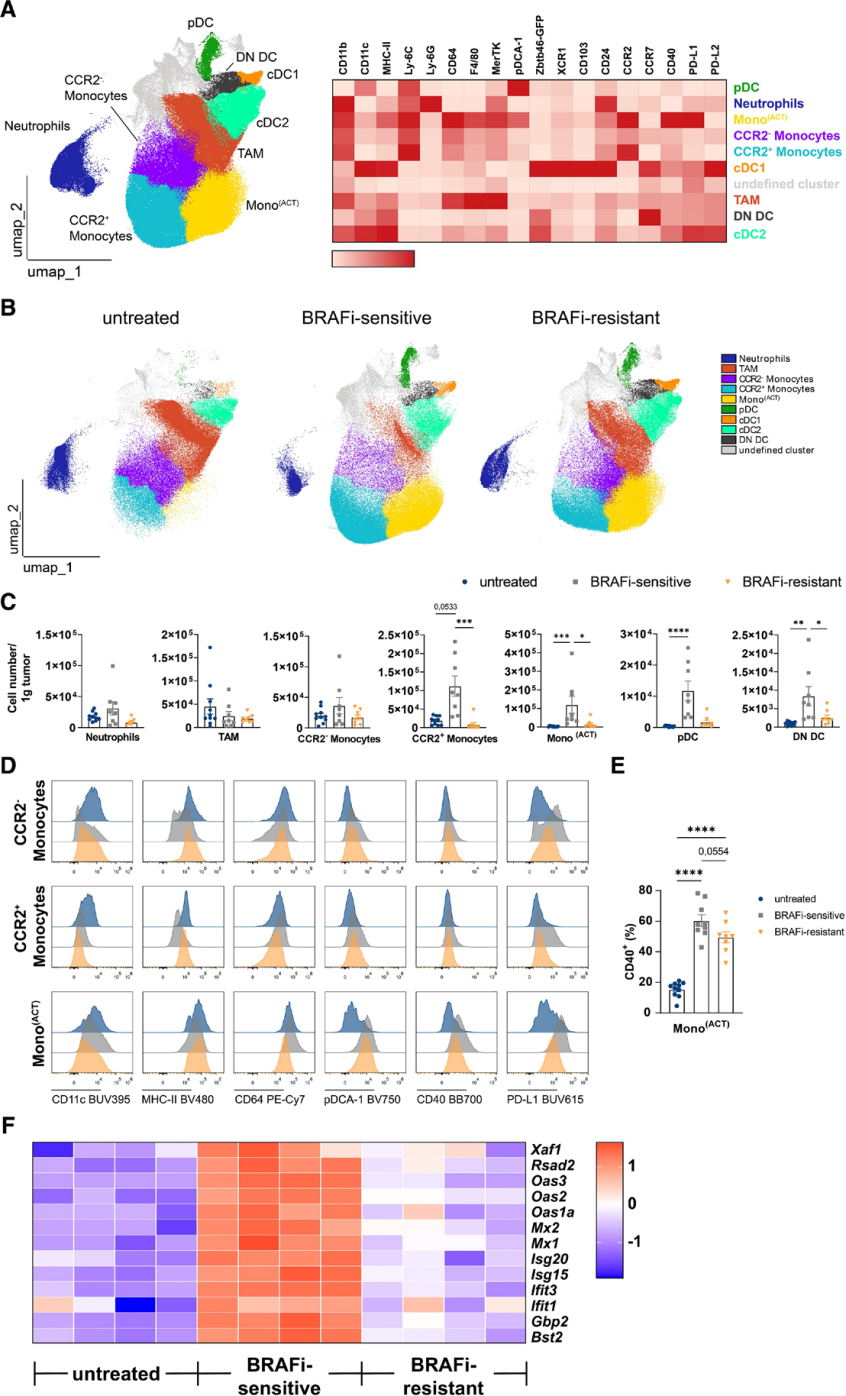
BRAFi treatment profoundly remodels the myeloid landscape in tumors. (A) Flow cytometry data from untreated, BRAFi-sensitive and -resistant tumors derived from Zbtb46^GFP/WT^ mice was concatenated. FlowSOM unsupervised clustering of viable CD45^+^ CD3^−^ NK1.1^−^ CD19^−^ myeloid cells. Left: UMAP of all cells and all groups (untreated, BRAFi-sensitive and BRAFi-resistant tumors). Right: heatmap displaying the expression of several myeloid markers on identified clusters across all three groups. (B) UMAPs of each treatment group showing three representative mice per group, displaying the changes in frequencies of the different identified myeloid cell clusters in tumors. (C) Cell numbers of tumor-infiltrating myeloid subtypes per gram tumor tissue in untreated, BRAFi-sensitive and BRAFi-resistant tumors. (D) Representative histograms showing the surface expression of several myeloid markers on moncytes subsets. (E) Percentages of CD40^+^Mono^(ACT)^ in tumors. (F) The heatmap depicts normalized and relative expression levels (z-score) for several selected interferon-associated genes (n=4 mice/group). For (C,E) results from three independent experiments are shown (n≥8 mice/group). Statistical significance was determined using one-way analysis of variance followed by Tukey’s multiple comparison test or Kruskal-Wallis test followed by Dunn’s multiple comparison test. Graphs show the mean±SEM. *p<0.05; **p<0.01; ***p<0.001; ****p<0.0001. BRAFi, V-Raf murine sarcoma viral oncogene homolog B inhibitors; cDC, conventional DC; DC, dendritic cells; DN, double negative; MHC, major histocompatibility complex; pDC, plasmacytoid DC; PD-L1, Programmed Cell Death-Ligand 1; UMAP, Uniform Manifold Approximation and Projection; TAM, tumor-associated macrophages.

Given the heterogeneity of the myeloid cell compartment, we decided to perform an unbiased, clustering analysis of the D4M tumor flow cytometry data using FlowSOM.^41^ For analysis we excluded dead cells, NK, NKT, T and B cells and used the myeloid cell gate (see online supplemental figure 1d). We identified 10 clusters that were projected on a UMAP space and all of them were assigned to a specific myeloid subset according to their surface marker expression. We characterized neutrophils, TAM, three monocyte clusters (CCR2^−^ monocytes, CCR2^+^ monocytes and Mono^(ACT)^), and four DC clusters (pDC, cDC1, cDC2 and double negative (DN) DC) (figure 3A). Neutrophils (cluster 1) were identified by their expression of Ly-6C as well as high levels of Ly-6G and CD11b. Based on the expression of F4/80, FcγRI/CD64 and MerTK, cluster 2 was identified as TAM. All monocyte clusters (3, 4 and 5) expressed Ly-6C and CD11b. CCR2^+^ monocytes had higher levels of Ly-6C than CCR2^−^ monocytes. The Mono^(ACT)^ subset expressed the inflammatory chemokine receptor CCR2 in addition to major histocompatibility complex (MHC)-II and CD64. Moreover, Mono^(ACT)^ also displayed activation markers such as CD40 and Programmed Cell Death-Ligand 1 (PD-L1) as recently described^42^ (figure 3A).

For the identification of the DC clusters, we made use of Zbtb46^GFP/WT^ reporter mice as this is a transcription factor selectively expressed by cDC.^32^ As expected, the three cDC clusters were Zbtb46-GFP positive, whereas pDC were clearly negative. Furthermore, cDC1 and cDC2 expressed high levels of CD11c and MHC-II. The cDC1 subset was identified by the exclusive expression of XCR1, whereas pDC expressed Ly-6C and pDCA-1. The DN DC were also positive for Zbtb46-GFP, but were characterized by the absence of XCR1 and CD11b. We also identified a cluster that we were not able to assign to a specific myeloid subset. These cells did not express CD11b, CD11c or MHC-II, lacked the expression of monocyte and macrophage markers and were negative for Zbtb46-GFP, thus we named them undefined cluster (figure 3A).

Tumor-targeted therapy with BRAFi had a strong impact on the myeloid immune cell compartment in the TME, as visualized in the UMAP space with many myeloid clusters shifting in relative frequency (figure 3B). When we calculated the absolute numbers of myeloid subsets in the tumor, neutrophils, TAM and CCR2^–^ monocytes were not significantly changed by BRAFi treatment (figure 3C). In line with the T cell-inflamed TME, we detected a transient infiltration of inflammatory CCR2^+^ monocytes, Mono^(ACT)^, pDC and DN DC on tumor-targeted therapy that was abrogated in resistant tumors (figure 3C).

To better define these inflammatory monocyte subtypes, we had a closer look at the expression levels of several myeloid markers. Mono^(ACT)^ displayed higher levels of CD11c, MHC-II and FcγRI/CD64 compared with CCR2^–^ and CCR2^+^ monocytes resembling monocyte-derived DC (moDC) that have been described in highly inflammatory conditions in vivo.^43 44^ Even more, Mono^(ACT)^ had higher levels of pDCA-1 (Bst-2) upon BRAFi therapy, a marker known to be induced by type I interferon (IFN)^45^ (figure 3D). In addition, Mono^(ACT)^ from BRAFi-treated tumors expressed higher levels of CD40, indicating a more activated phenotype (figure 3E). In line with these findings, RNA-seq analysis of tumors revealed an upregulation of several genes involved in IFN signaling during the BRAFi-sensitive phase. Among these, we observed IFN-stimulated genes (ISG), including *Isg15, Isg20, Mx1, Mx2 or Bst2* (figure 3F).

Our in-depth flow cytometry analysis of the myeloid compartment highlights the complexity of myeloid subtypes and the high plasticity of monocytic cells. In fact, tumor-targeted therapy with BRAFi profoundly remodels the myeloid immune compartment in the TME with a transient accumulation of inflammatory monocytes and pDC including the recently described Mono ^(ACT)^ subtype resembling moDC by their marker expression. Collectively, the changes in the myeloid compartment mirror the induction of a T cell-inflamed TME early on during tumor-targeted therapy that reverts to an immunological inert milieu during resistance development.

### Activated migratory cDC1 and cDC2 infiltrate BRAFi-treated tumors

**Figure 4:**
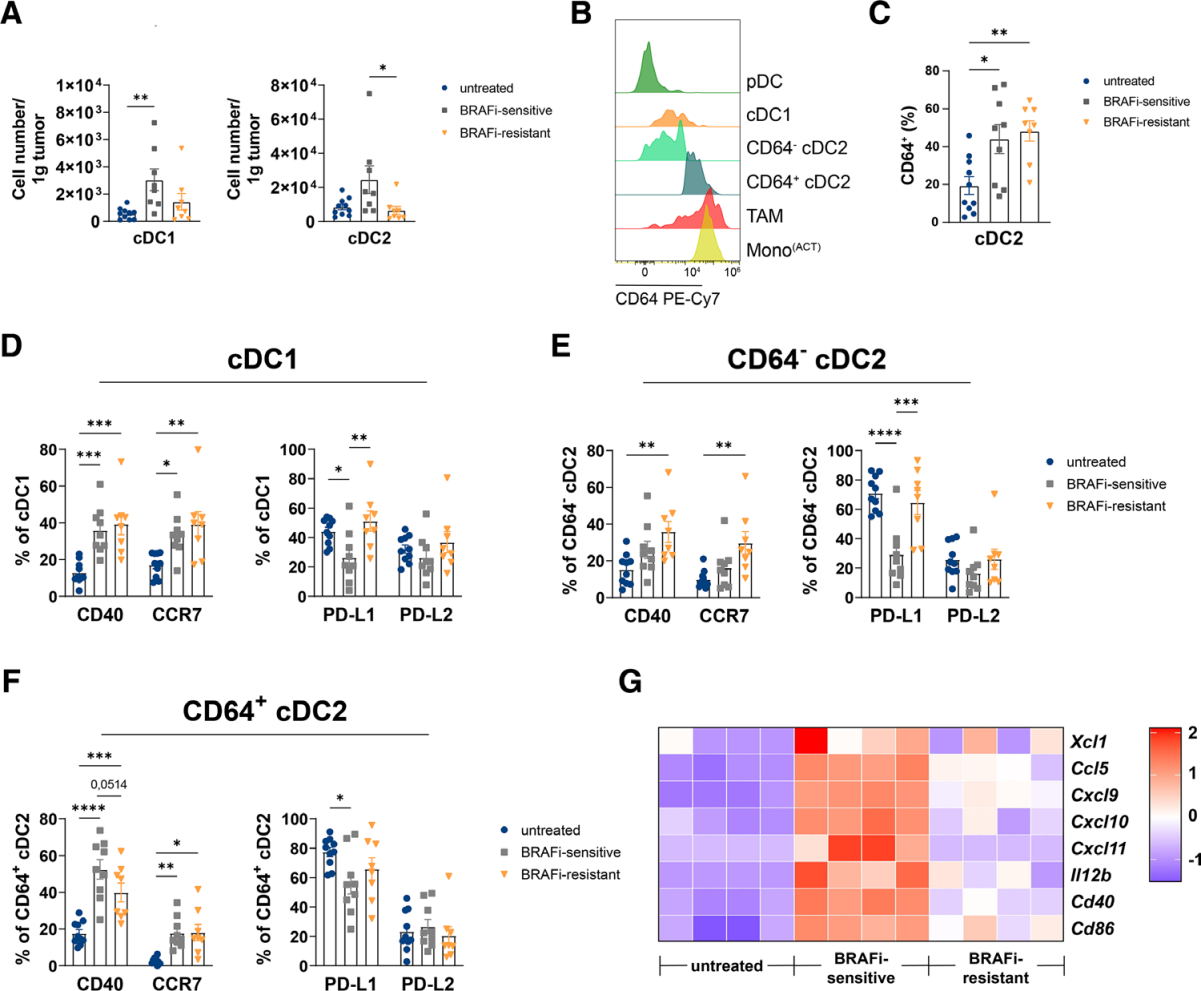
BRAFi treatment causes a transient infiltration of activated DC subtypes to D4M tumors. (A) Cell numbers of cDC1 and cDC2 per gram tumor tissue from untreated, BRAFi-sensitive and BRAFi-resistant tumors are shown. (B) Representative histogram for CD64 expression on the different myeloid cell clusters in D4M tumors. (C) Percentages of CD64^+^ cDC2. (D–F) Summary graphs depicting percentages of CD40, CCR7, PD-L1 and PD-L2 positive cDC1, CD64^–^ cDC2 and CD64^+^ cDC2. (G) RNA sequencing analysis of D4M tumor tissue from untreated, BRAFi-sensitive and BRAFi-resistant tumors. The heatmap depicts normalized and relative expression levels (z-score) of several DC-related cytokines and chemokines (n=4 mice/group). For (A,C–F) results from three independent experiments are shown (n≥8 mice/group). Statistical significance was determined using one-way analysis of variance followed by Tukey’s multiple comparison test or Kruskal-Wallis test followed by Dunn’s multiple comparison test. Graphs show the mean±SEM. *p<0.05; **p<0.01; ***p<0.001. BRAFi, V-Raf murine sarcoma viral oncogene homolog B inhibitors; cDC, conventional DC; DC, dendritic cells; pDC, plasmacytoid DC; PD-L1, Programmed Cell Death-Ligand 1; TAM, tumor-associated macrophages.

Our results so far show that tumor-targeted therapy profoundly shapes the myeloid landscape. Thus, we studied in more detail how BRAFi impacts cDC1 and cDC2 subsets due to their crucial role in tumor immunity.^20 26 29 30^ As mentioned above, we used Zbtb46^GFP/WT^ mice as a cDC-specific reporter mouse strain^32^ to discriminate DC from TAM and monocytes as they share surface marker expression, particularly in tumors. From our FlowSOM analysis of the myeloid cell compartment, we could define the main cDC subsets. After the identification of cDC by means of Zbtb46-GFP expression (figure 3A), we characterized cDC1 by their surface staining for XCR1 and cDC2 by CD11b (figure 3A, online supplemental figure 3a,b). The overall number of cDC1 and cDC2 in tumors was increased in D4M tumors early on during BRAFi therapy and their frequency went down during resistance development (figure 4A) reflecting the T cell-inflamed phenotype induced by BRAFi therapy.

Interestingly, flow cytometry analysis of tumors revealed the appearance of cDC2 expressing the FcγRI/CD64 (online supplemental figure 3c), a marker often used to distinguish cDC from monocytes.^46^ However, as these cells were expressing Zbtb46-GFP, we assume that they have a pre-DC origin. Without the use of Zbtb46^GFP/WT^ mice it would be very difficult to separate these cells from other myeloid cells. The intensity of FcγRI/CD64 expression on CD64^+^ cDC2 was between that of cDC and TAM/Mono^(ACT)^ (figure 4B). More CD64^+^ cDC2 were detected in tumors with the onset of tumor-targeted therapy that remained abundant in resistant tumors (figure 4C). Remarkably, the CD64^+^ cDC2 displayed higher levels of the DC activation marker CD40 shown as median fluorescent intensity (MFI), compared with CD64^−^ cDC2 and cDC1, when analyzed across all three different tumor stages. In contrast, the cDC1 subset displayed the highest levels for the chemokine receptor CCR7 indicating their migratory potential (online supplemental figure 3d).

A hallmark of DC is their high functional plasticity. Depending on the context, they can be either immunostimulatory or immunosuppressive. Interestingly, all DC subsets downregulated the inhibitory molecule PD-L1 during the BRAFi-sensitive phase. In the same way, cDC1 and especially CD64^+^ cDC2 strongly upregulated CD40 and CCR7 expression indicative of the acquisition of an activated migratory phenotype in response to BRAFi therapy. Interestingly, the upregulation of CD40 and CCR7 is more pronounced on CD64^+^ cDC2 compared with CD64^–^ cDC2 (figure 4D–F). In line with the enhanced infiltration of DC, RNA-seq analysis revealed in BRAFi- sensitive tumors an upregulation of several genes involved in DC recruitment and DC-T cell interaction, such as inflammatory chemokines. For example, *Xcl1* and *Ccl5* mediate cDC1 infiltration into tumors,^47^ whereas *Cxcl9* and *Cxcl10* are important for DC-T cell interaction by recruitment of CXCR3^+^ effector T cells^48 49^ and for effective T cell-mediated melanoma growth control.^25^ Moreover, the genes for DC-derived cytokine *Il12* for induction of Th1 response and costimulatory molecules for T cell stimulation *Cd40* and *Cd86* were strongly upregulated during BRAFi-sensitive phase and downregulated in resistant tumors (figure 4G).

In summary, alterations in the frequency of cDC1 and cDC2 subsets and their activation profile follow a similar scheme to the earlier described T cell response during BRAFi therapy. The highly inflamed TME contains an additional activated CD64^+^ DC subtype which together with CD40^+^ CCR7^+^ cDC1 and cDC2 are well equipped to drive a T cell response.

### BRAFi therapy drives the migration of activated DC subsets to tumor-draining lymph nodes

So far we demonstrated that BRAFi therapy strongly impacts the immune infiltrate in tumors, in favor of cytotoxic effector T cells, activated monocytes and DC early on during treatment. As de novo T-cell responses are initiated in tumor-draining LN, we next investigated DC subsets and their activation profile in the lymphatic tissue. We decided to identify the different DC populations by manual gating as they are well-defined in LN.^38^

**Figure 5:**
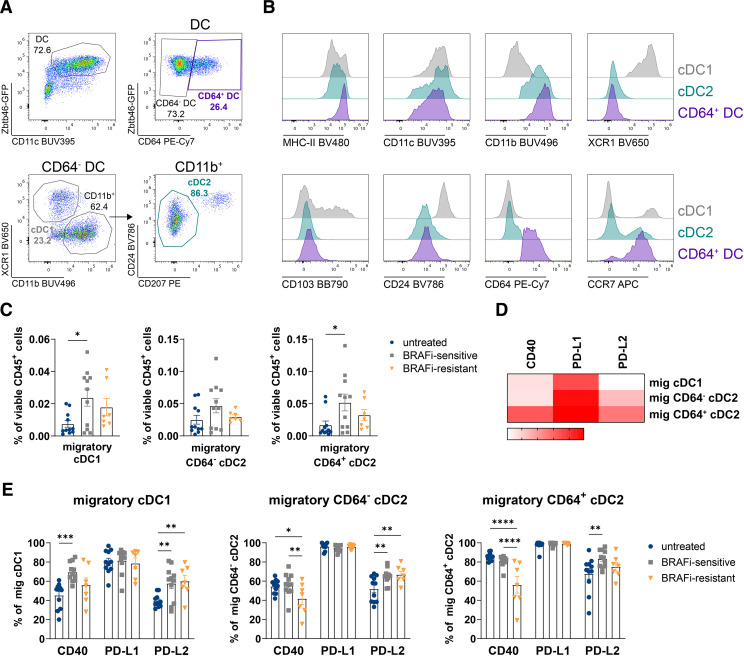
BRAFi increases the percentage of activated, migratory DC in tumor-draining LN. (A) Representative flow cytometry plots for the identification of CD64^+^ DC, cDC1 and cDC2 after exclusion of dead cells, natural killer cells, NKT cells, T cells, B cells, pDC, monocytes and neutrophils (for gating strategy see online supplemental figure 4a). Zbtb46-GFP and CD11c expression was used for correct DC-discrimination from other myeloid cell populations. (B) Representative histograms showing surface expression of myeloid markers on the different DC populations in tumor-draining LN. (C) Percentages of migratory DC subsets in tumor-draining LN of untreated, BRAFi-sensitive and BRAFi-resistant tumors. (D) Heatmap displaying the percentage of CD40, PD-L1 and PD-L2 expression on migratory DC. (E) Phenotypical characterization of migratory DC subsets in tumor-draining LN by analyzing the expression of CD40, PD-L1 and PD-L2. For (C–E) results from three independent experiments are shown (n≥7 mice/group). Statistical significance was determined using one-way analysis of variance followed by Tukey’s multiple comparison test or Kruskal-Wallis test followed by Dunn’s multiple comparison test. Graphs show the mean±SEM. *p<0.05; **p<0.01; ***p<0.001; ****p<0.0001. BRAFi, V-Raf murine sarcoma viral oncogene homolog B inhibitors; cDC, conventional DC; DC, dendritic cells; LN, lymph nodes; mig DC, migratory DC; NKT, natural killer T; pDC, plasmacytoid DC; PD-L1, Programmed Cell Death-Ligand 1.

After identification of DC by Zbtb46-GFP and their CD11c expression, we observed that similar to D4M tumors, a considerable proportion of DC in tumor-draining LN expressed FcγRI/CD64, which has been described as a monocytic/macrophage marker.^46 50^ Besides this CD64^+^ DC we split the CD64^−^ DC into XCR1^+^ cDC1 and CD11b^+^ cDC2 (figure 5A). More detailed phenotypical analysis of the CD64^+^ DC revealed the expression of CD11b but a lack of XCR1, CD103 and CD24 expression, indicating their relationship to the cDC2 subset. Furthermore, we realized that a high number of those cells was positive for CCR7 suggesting a migratory phenotype (figure 5B). With this knowledge, we established a gating strategy that allowed us to identify total resident LN-DC and migratory cDC1, cDC2 and CD64^+^ cDC2 by using the chemokine receptor CCR7. As Langerhans cells (LC) do not infiltrate the transplantable tumors, we excluded LC in tumor-draining LN from further analysis (online supplemental figure 4a,b).

BRAFi therapy increased proportions of all migratory LN-DC subsets, especially cDC1 and CD64^+^ cDC2, although CD64^−^ cDC2 to a lesser extent, and these percentages just slightly decreased in the resistant phase (figure 5C). In contrast, the abundance of resident DC and pDC in tumor-draining LN was not affected by BRAFi treatment (online supplemental figure 4c,d).

The CD64^+^ cDC2 displayed the highest MFI of the DC activation marker CD40 in tumor tissue (online supplemental figure 3d). This is reflected in the tumor-draining LN, as almost all migratory CD64^+^ cDC2 were CD40^+^ positive indicating that they were highly activated (figure 5D). When we characterized the DC subsets during BRAFi therapy, more CD40^+^ activated, migratory cDC1 arrived in tumor-draining LN during BRAFi-sensitive phase, whereas percentages of CD40^+^ migratory CD64^–^ cDC2 and CD64^+^ cDC2 were unchanged. Interestingly, all three DC subsets downregulated CD40 expression during BRAFi-resistant phase. PD-L1 and PD-L2 molecules have been described as activation markers on migratory DC.^51^ PD-L1 was detected on all DC subsets to a high extent without any changes during BRAFi therapy, in contrast, PD-L2 was upregulated on all DC subsets with the onset of therapy (figure 5E).

CD64-expressing cDC2 are not restricted to the BRAF-mutant D4M tumor model as this DC subtype could also be detected in the melanoma mouse model B16-ovalbumin (OVA) in the tumor tissue but also tumor-draining LN (online supplemental figure 5). An intriguing observation was that CD64^+^ cDC2 were almost absent in skin-draining inguinal LN of tumor-free mice (online supplemental figure 5c), suggesting that CD64-expression seems to be triggered by tumor-derived factors.

Our observations reveal that during tumor-targeted therapy more activated, migratory DC end up in tumor-draining LN, an important location for initiation of de novo T-cell responses. During resistance development against BRAFi the percentages of activated migratory DC decreased, pointing at impaired T cell priming in tumor-draining LN.

### Resistance development to tumor-targeted therapy impairs T-cell responses in tumor-draining LN

**Figure 6:**
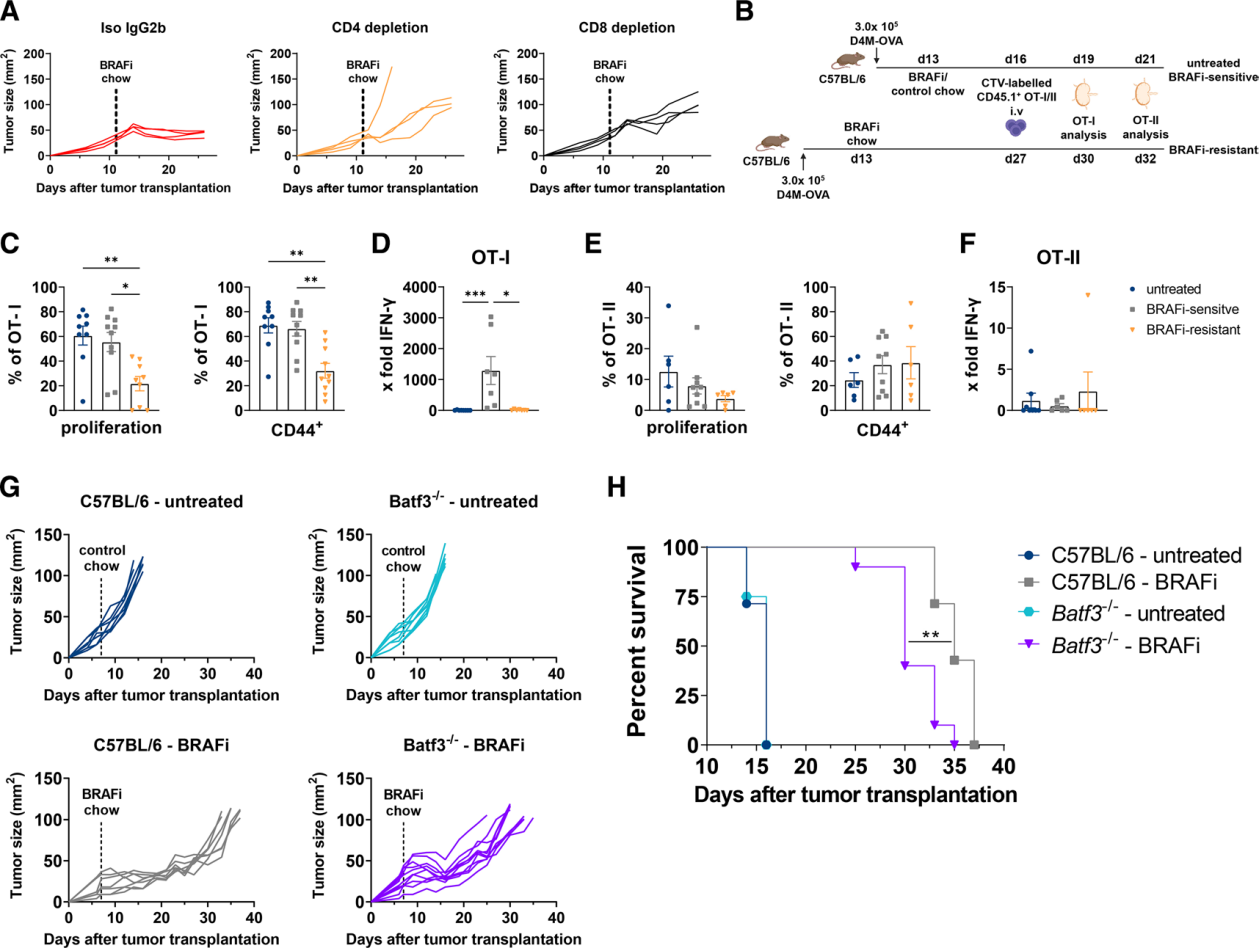
Resistance to tumor-targeted therapy impairs antitumor CD8^+^ T-cell response. (A) Individual D4M tumor growth in CD4^+^ or CD8^+^ T-cell depleted C57BL/6 mice during BRAFi treatment compared with isotype control. (B) Experimental design of the in vivo antigen-specific T-cell assay. 3×10^5^ D4M-OVA melanoma cells were s.c. injected into the flank skin of C57BL/6 mice. CTV-labeled CD4^+^ OT-II or CD8^+^ OT-I T cells were adoptively transferred into separate untreated, BRAFi-sensitive and BRAFi-resistant animals. Proliferation and activation of antigen-specific CD8^+^ T cells was analyzed by flow cytometry 3 days after transfer, antigen-specific CD4^+^ T cells were analyzed 5 days after transfer. (C) Percentages of proliferating and CD44^+^ antigen-specific OT-I T cells in the tumor-draining LN. (D) Results for IFN-y ELISA after in vitro restimulation of tumor-draining LN cells with 1 µM OVA_257-264_ peptide for 48 hours. (E) Percentages of proliferating and CD44^+^ antigen-specific OT-II T cells in the tumor-draining LN. (F) Results for IFN-y ELISA after in vitro restimulation of tumor-draining LN cells with 1 µM OVA_323-339_ peptide for 48 hours. (G,H) D4M tumor growth in Batf3^−/−^ or C57BL/6 mice. Individual D4M tumor growth (G) and Kaplan-Meier survival curve (H) is shown (n≥7/group). For (C–F) results from two independent experiments are shown (n≥6 mice/group). For (D,F) the fold change over negative controls is shown. As negative controls, CTV-labeled CD4^+^ OT-II or CD8^+^ OT-I T cells were adoptively transferred into separate tumor-free C57BL/6 mice. Statistical significance was determined using one-way analysis of variance followed by Tukey’s multiple comparison test or Kruskal-Wallis test followed by Dunn’s multiple comparison test. Graphs show the mean±SEM. *p<0.05; **p<0.01; ***p<0.001.BRAF, V-Raf murine sarcoma viral oncogene homolog B inhibitors; CTV, CellTrace Violet; IFN, interferon; LN, lymph nodes; OVA, ovalbumin.

In a final step, we investigated the importance of T-cell responses during tumor-targeted therapy to understand better how the T cell-inflamed TME mediates therapy success with BRAFi. For this purpose, we depleted CD4^+^ or CD8^+^ T cells in D4M tumor-bearing mice by injection of specific antibodies (online supplemental figure 6a,b). Depletion of CD8^+^ T cells drastically shortened the time of BRAFi-mediated tumor control to 1 week, whereafter the tumors progressed quickly to maximum size. Interestingly, mice depleted of CD4^+^ T cells also demonstrated earlier resistance development to BRAFi therapy indicating an essential contribution of CD4^+^ T cells to tumor regression caused by BRAFi treatment (figure 6A).

To comprehensively characterize tumor antigen-specific CD8^+^ and CD4^+^ T-cell responses, we first generated an OVA-protein transduced D4M cell line. D4M-OVA cells demonstrated a slightly delayed tumor growth due to the higher immunogenicity of the OVA-transgene (online supplemental figure 6c). Thus, BRAFi treatment was started on day 13 instead of day 8 whereafter D4M-OVA tumors responded with the same sensitivity to BRAFi treatment as the parental D4M cell line. Untreated mice reached maximum D4M-OVA tumor size within 19 days after transplantation. Treatment with BRAFi led to a reduction in D4M-OVA tumor size within 1 week of treatment and tumor growth was controlled for 2–3 weeks before resistance developed (online supplemental figure 6c).

For in vivo T-cell assays, we transferred fluorescently labeled CD45.1 OVA-specific OT-I or OT-II T cells into D4M-OVA bearing CD45.2^+^ mice and investigated their proliferation and activation in tumor-draining LN (figure 6B). Although we observed no additional impact of BRAFi therapy over untreated mice on CD8^+^ T-cell proliferation and activation most likely due to the high immunogenicity of D4M-OVA tumors, we observed a significant decrease of OVA-specific CD8^+^ T-cell proliferation in the BRAFi-resistant group. This was also reflected by an impaired activation of OVA-specific CD8^+^ T cells, indicated by lower expression levels of CD44 (figure 6C). LN cell suspensions were restimulated in vitro with OVA_257-264_ peptide and levels of released IFN-γ were measured via ELISA. Despite high proliferation and activation rates, OT-I T cells from untreated animals failed to release IFN-γ, arguing that they are dysfunctional. In the BRAFi-sensitive phase with its highly inflamed TME, OT-I T cells in tumor-draining LN produced high amounts of IFN-γ, and this effector function was completely lost when BRAFi-resistance developed (figure 6D). The proliferation and activation of OVA-specific CD4^+^ T cells was way lower than OT-I and showed no clear differences (figure 6E). This is reflected by the absence of IFN-γ in supernatants of OVA_323-339_ peptide restimulated OT-II T cells (figure 6F) demonstrating that the tumor model antigen OVA was mainly presented to CD8^+^ T cells.

As we observed a tumor antigen-specific CD8^+^ T-cell response in the early phase of BRAFi therapy and cDC1 are reported to be the best cross-presenting DC subset, we wanted to assess the contribution of cDC1 to T cell immunity during BRAFi therapy. To determine if cDC1 are essential for the efficacy of BRAFi treatment, we transplanted D4M cells into Batf3^−/−^ mice lacking cDC1 and C57BL/6 wildtype mice as control. First, we confirmed the absence of cDC1 during BRAFi therapy course by investigating cDC1 in spleens of tumor-bearing mice (online supplemental figure 6d). Interestingly, in Batf3^−/−^ mice tumor-targeted therapy with BRAFi still worked by controlling tumor growth comparable to C57BL/6 mice, however, resistance developed earlier (figure 6G). The survival curves revealed that Batf3^−/−^ mice had a worse outcome than C57BL/6 mice, suggesting that BRAFi-mediated antitumor immunity partially depends on cDC1 but a contribution by cDC2 to T cell immunity is quite likely (figure 6H).

Overall we conclude that T cell immunity is crucial for the efficacy of tumor-targeted therapy with BRAFi as selective depletion of T cell subsets compromised therapy success. In addition, adoptively transferred CD8^+^ T cells were only capable of IFN-γ production in LN draining BRAFi-sensitive tumors with the T cell-inflamed TME harboring activated and migratory DC. As soon as resistance develops the antitumor CD8^+^ T-cell response is impaired reflecting the inert state of the TME.

## Discussion

In our study, we reveal that tumor-targeted therapy with BRAFi not only creates a T cell-inflamed TME, but also shapes the myeloid landscape in tumors. Inflammatory monocytes and DC subsets cDC1 and cDC2 including a subpopulation expressing the FcγRI/CD64 emerged in BRAFi-treated tumors with a highly activated phenotype. The functional properties of tumor antigen-specific CD8^+^ T cells in the draining LN were boosted by BRAFi, however, cDC1 were not alone in driving T cell immunity. During resistance development tumors became immunologically inert with a loss of activated monocytes and DC combined with an accumulation of Tregs. Overall, our findings give novel insights into the importance of myeloid cells in tumor-targeted therapy, which might open new avenues for future combinatorial treatment options with DC therapy.

DC play an important role in orchestrating T cell-mediated antitumor immunity and are crucial for the success of immunotherapy.^22^ Indeed, previous studies have clearly demonstrated that DC are essential for the efficacy of immune checkpoint blockade.^30 52^ A recent study demonstrated that acquired resistance to tumor-targeted therapy with MAPK pathway inhibitors leads to the development of cross-resistance to immunotherapies. This cross-resistance was characterized by a lack of functional CD103^+^ DC,^53^ however, detailed analysis of other myeloid cell types and DC subsets over the time course of BRAFi therapy was not performed. Therefore, the aim of our study was to investigate the myeloid cell compartment with a focus on DC subtypes during tumor-targeted therapy with BRAFi. We used the transplantable BRAF^V600E^-mutant D4M.3A melanoma mouse model that demonstrated a robust response to BRAFi treatment for approximately 2–3 weeks before resistance developed. This makes the D4M.3A melanoma cell line a highly suitable preclinical model to investigate immunological effects in a timed way during the different phases of BRAFi therapy similar to the patient studies performed so far.^11 54^

Early on during BRAFi therapy a transient remodeling process of the lymphoid and myeloid landscape took place with a recruitment of CCR2^+^ inflammatory monocytes. With our RNA-seq data analysis we identified the upregulation of several genes involved in IFN signaling which can drive the activation of monocytes as recently reported.^42^ The chemokine CCL2 essential for the attraction of inflammatory monocytes to tissue^55^ as well as the chemokines XCL1 and CCL5 important for the recruitment of cDC1 to tumor tissue^47^ were upregulated during the BRAFi therapy as shown here and by our previous work.^17^ This is most likely mediated by the induction of a distinct form of immunogenic cell death termed pyroptosis as shown by us in this study here and by others previously.^33^ Mechanistically, MAPK inhibition leads to caspase-3 cleavage subsequently inducing cleavage of GSDME causing pore formation in the plasma membrane. Through this pores damage-associated molecular patterns, such as HMGB1 and HSP90 can be released, which is known to activate DC via Toll-Like Receptor (TLR)4 leading to the promotion of T cell-mediated antitumor immunity.^35 56^

As DC are crucial for the induction of antitumor immunity,^22^ we focused on the changes in DC subset distribution during tumor-targeted therapy. Our study provides clear evidence that tumor-targeted therapy with BRAFi affects DC trafficking into tumors. These observations are in line with earlier studies with RNA-seq data analysis from patient samples on tumor-targeted therapy. The deconvolution of bulk RNA-seq data to quantify immune cell types from pretreatment and on-treatment melanoma biopsies of patients under BRAFi therapy revealed an infiltration of DC on treatment together with T cell accumulation similar to our observations.^54^ Another study analyzed a publicly available RNA-seq data set of BRAFi-treated patient tumors and highlighted a gene signature associated with increased DC infiltration when tumors were responding to the treatment.^33^ With our detailed analysis of myeloid subtypes in BRAFi-treated D4M tumors, we here report the transient recruitment of both DC subsets cDC1 and cDC2 to BRAFi-treated tumors with a prominent subpopulation of FcγRI/CD64-expressing cDC2. The tumor-infiltrating cDC1 and cDC2 displayed a migratory and activated phenotype shown by CD40 and CCR7 expression, the RNA-seq data revealed overall upregulation of *Cd40*, *Cd86* but also the cytokine *Il12* in BRAFi-sensitive tumors. Interestingly, the CD64^+^ cDC2 showed the highest levels of costimulatory molecule CD40 expression arguing that CD64 could be a biomarker for the presence of activated DC.

The central role of cDC1 in antitumor immunity and in cancer therapy is well documented.^18 19 25 53^ One crucial feature is the production of the chemokine CXCL9 by cDC1 in the TME facilitating the formation of DC-T cell clusters and cross-presentation of tumor antigens within those niches, which is essential for functional aspects of intratumoral CD8^+^ T cells and tumor immune control.^26^ In our study, the absence of cDC1 in Batf3-deficient mice shortened the survival of mice in response to BRAFi therapy but did not cause a complete failure of treatment. This suggests a potential role of cDC2 in the immune response mediated by BRAFi, as cDC2 can promote antitumor immunity. Using a transgenic melanoma mouse model, our group previously demonstrated that migratory skin cDC2 cross-present tumor antigens to CD8^+^ T cells.^30^ Another report confirmed that cDC2 are essential for CD4^+^ T cell responses in tumor models.^29^ Interestingly, D4M tumors contained cDC2 expressing the FcγRI/CD64 as confirmed by the Zbtb46^GFP/WT^ reporter mouse model that allowed to distinguish CD64^+^ cDC2 from monocyte-derived cells^46^ and macrophages.^50^ Similar DC subtypes have been described in infection, for example, with *Listeria* or respiratory virus infection. The differentiation of those CD64-expressing cDC2, termed inflammatory cDC2 type is driven by type I IFN. They are proficient in inducing CD4^+^ T helper cell polarization but they are also capable of efficiently presenting soluble but also immune complexed antigens to CD8^+^ T cells.^43 57^ In a tumor regression model, CD11b^+^ cDC2 that displayed an IFN-stimulated gene expression (ISG^+^ DC) could present intact tumor-peptide-MHC-I complexes via cross-dressing.^58^ As we observed an upregulation of several genes involved in type I IFN signaling during BRAFi-sensitive phase, we assume that the CD64^+^ cDC2 population we identified resemble this inflammation-induced DC subtype.

Although LC do not infiltrate the D4M tumor model, it is well established that LC can present antigens derived from the skin to CD4^+^ and CD8^+^ T cells in the draining LN.^59^ LC are localized in the epidermis next to melanoma, therefore playing an important role in antitumor immunity.^60^ In fact, we previously demonstrated that LC can cross-present gp100 tumor-associated antigen in tumor-draining LN, although to a lesser extent compared with cDC1 and cDC2.^30^

The occurrence of activated cDC1, cDC2 including the CD64^+^ DC subset coincided with the accumulation of activated GrzB-positive CD4^+^ and CD8^+^ T cells in BRAFi-treated tumors. We confirmed with our RNA-seq data the upregulation of genes for cytotoxic mediators, for example, *Gzmb, Prf1, Fasl*, and chemokines for attraction of activated T cells, for example, *Cxcl9, Cxcl10, Cxcl11*. These type I IFN-induced chemokines are essential for DC-T cell interaction locally in tumor tissue.^61^ The infiltration of activated cytotoxic T and NK cells during BRAFi therapy has been demonstrated in patients with melanoma^9–11^ but also in various melanoma mouse models.^13–15 17^ However, our study now provides novel insights into the localization of tumor-infiltrating effector T cells by multiplex imaging. The inflammation induced by BRAFi recruits cytotoxic GrzB^+^ CD4^+^ and CD8^+^ T cells right into the tumor center away from the tumor margin where T cells are localized in untreated tumors. Interestingly, not just CD8^+^ T cells but also CD4^+^ T cells were GrzB^+^, a feature reported earlier in the context of cancer,^62^ and the role of CD4^+^ T cells in antitumor immunity receives increasing attention.^29 63^ The frequency of cytotoxic CD4^+^ and CD8^+^ T cells in BRAFi-treated tumors was reduced on development of resistance. Moreover, tumor-infiltrating Treg outnumbered CD8^+^ T cells in resistant tumors arguing for a more immunosuppressive TME. The relevance of T cell immunity for the success of tumor-targeted therapy was proven by the depletion of CD8^+^ T cells or CD4^+^ T cells in our tumor model as their absence abrogated the efficacy of BRAFi treatment. Our findings are in line with studies on mouse models reporting that a loss of intratumoral T cells is associated with resistance to tumor-targeted therapy using BRAFi in combination with MEK inhibitors.^33 64^ The importance of T cell-antigen-presenting cell interactions at the tumor invasive margins is indicated by the observation that CD4^+^ effector T cells have the ability to promote the recruitment and IFN-dependent activation of monocytes.^63^ In support of this, monocytes are crucial for the recruitment and spatial organization of T cells^65^ and can promote effector CD8^+^ T cell function.^42^ The aforementioned studies suggest that the absence of T cells during BRAFi therapy in our model might impair recruitment of activated monocytes or DC to the TME consequently leading to a failure to elicit a robust antitumor CD8^+^ T cell response.

A crucial step in the induction of antitumor immunity is the priming of T cells in the tumor-draining LN. Migratory cDC1 and cDC2 are potent cross-presenters of tumor antigens in LN.^30 66^ In our study, we observed higher frequencies of activated migratory cDC1 and migratory cDC2, which contained again a large proportion of CD64-expressing cells on BRAFi therapy. Again, the CD64^+^ DC subset showed the highest levels of CD40 making them interesting targets for modulation of T cell responses. The IFN-γ production in adoptively transferred tumor-specific CD8^+^ T cells was boosted by BRAFi treatment, which can be well explained by the favorable inflammatory TME that enables the migration of activated DC to the draining LN. In contrast, during resistance development to BRAFi, the percentages of activated migratory DC subsets decreased and in vivo CD8^+^ T cell response in tumor-draining LN was impaired. The exact role of this CD64^+^ cDC2 subset in tumor immunity needs to be confirmed in future studies, as no suitable CD64^+^ DC-depletion mouse model is currently available. An additional shortcoming of our study is the lack of primary patient samples to perform flow cytometry analysis of DC subsets in patients with BRAFi-single treated melanoma.

In conclusion, our study provides novel knowledge on the immune modulation occurring during tumor-targeted therapy with BRAFi leading to a T cell-inflamed TME characterized by the infiltration of cytotoxic T cells, inflammatory monocytes and activated cDC1 and cDC2. As the most activated migratory cDC2 displayed the FcγRI/CD64 on their surface, this could be a potential biomarker for immune reactive DC. On resistance development, the tumors revert to an immunologically inert milieu with the loss of inflammatory monocytes, activated DC, effector T and NK cells and recruitment of potentially immunosuppressive Treg. We here describe the complexity of the myeloid network in tumors that needs to be considered in future studies by developing optimized antibody panels for high-dimensional flow cytometry and microscopy to decipher the cellular network in tumor tissue. In this regard, the role of the cDC2 population in antitumor immunity needs more attention to harness their potential for future immunotherapies. Our findings have implications for the careful timing of tumor-targeted therapy with immunotherapy to benefit from the highly immunogenic milieu early on during tumor-targeted therapy. More DC-based approaches by boosting DC numbers and activation with Flt3L and anti-CD40 antibodies^19 30 53^ could be worthwhile exploring, instead of checkpoint blockade antibodies which proved highly toxic in combination with BRAF/MEK inhibition.^67–69^

## Materials and methods

### Mouse models

Zbtb46^GFP/WT^ (obtained from Sandrine Henri, Centre d'Immunologie de Marseille-Luminy, Aix Marseille Université) express enhanced green fluorescent protein (EGFP) in all cDC subtypes and committed progenitors.^32^ Breeding pairs for C57BL/6 mice were purchased from Charles River Laboratories (Sulzfeld, Germany). OT-I and OT-II mice were on CD45.1 background and were kindly provided by Angelika Sales (Department of Biosciences, University of Salzburg). All animal experimental protocols were approved by the Austrian Federal Ministry of Science and Research (2021–0.117.131) and performed according to institutional guidelines. Zbtb46^GFP/WT^, C57BL/6N, OT-I and OT-II were housed and bred at the animal facility of the Department of Dermatology, Venereology and Allergology (Medical University of Innsbruck). Batf3^−/−^ mice lacking cDC1^27^ were housed and bred at the Biomedical Research Unit at the Malaghan Institute of Medical Research, Wellington, New Zealand.

### Mouse tumor cell lines

D4M.3A (D4M) murine melanoma cell line (derived from *Tyr::CreER; Braf* ^V600E^ *;Pten* ^−/−^ mice,^31^) was kindly provided by Constance E. Brinckerhoff (Geisel School of Medicine at Dartmouth, Norris Cotton Cancer Center, Lebanon, New Hampshire, USA). To generate D4M-OVA tumor cells for functional assays, D4M cells were transduced with lentivirus-expressing OVA-protein. The lentivirus was cloned by Gibson Assembly (NEB, Ipswich, USA). Briefly, the blasticidin encoding lentiviral expression backbone pLenti CMVie-IRES-BlastR (Addgene, plasmid #119863) was digested with PacI and NheI restriction enzymes (NEB). The protein-encoding sequence of the OVA transcript (NM_205152.3) was flanked with vector-overlapping sequences by PCR with the forward primer OVA_fwd (G T T T T G A C C T C C A T A G A A G A T T C T A G A G C T A G C G C C A C C A T G G G C T C C A T C G G C G C A G C A A G) and the reverse primer OVA_rev (G A G A G G G G C G G A T C C C C T T A A T T A A T C A A G G G G A A A C A C A T C T G C). Gibson assembly was performed at 50°C for 4 hours, followed by heat-shock transformation of 10-beta competent *Escherichia coli* (NEB) with the Gibson reaction mix. *E. coli* colonies on LB agar containing 50 µg/mL ampicillin (AppliChem, Illinois, USA) were subjected to colony-PCR with OVA_fwd primer and IRES_rev (G T G T G C G T T T G T C T A T A T G) primer binding to IRES downstream of the OVA insert. Positive *E. coli* clones were confirmed by Sanger sequencing (Microsynth, Balgach, Switzerland) using the OVA_rev and CMV_fwd (C G C A A A T G G G C G G T A G G C G T G) primers. Lentiviruses were produced through the process of CaPO_4_ transfection of HEK293T cells, in which the lentiviral plasmid was introduced along with GAG-PRO-POL and VSV glycoprotein. Lentivirus-containing supernatant from transfected HEK293T cells was filtered through a 0.2 µm filter, 1:5 diluted with Dulbeccos Modified Eagle Medium (DMEM) (Sigma-Aldrich, St. Louis, Missouri, USA) and supplemented with 8 µg/mL polybrene (Sigma-Aldrich). For spin infection, 2 mL of the supernatant was used to resuspend 5×10^5^ D4M cells. Cells were incubated for 20 min at room temperature (RT), plated into a 6-well plate and centrifuged at 625 g for 1 hour at 31°C. The cells were cultured for 48 hours to allow lentiviral DNA integration, and then selected with 10 µg/mL Blasticidin (InvivoGen, San Diego, California, USA). Single-cell clones were obtained by serial dilution in a 96-well plate. OVA-expression of the individual clones was verified by western blot analysis. D4M and D4M-OVA cells were cultured in DMEM (containing high glucose and L-glutamine, Sigma-Aldrich) supplemented with 5% heat-inactivated fetal calf serum (FCS; PAN-Biotech, Aidenbach, Germany), 50 U/mL penicillin and 50 µg/mL streptomycin (both Thermo Fisher Scientific, Waltham, Massachusetts, USA). B16-OVA tumor cells were cultured in IMDM medium (PAN-Biotech) supplemented with 10% FCS (PAN-Biotech), 50 µg/mL Gentamycin (Gibco, Paisley, UK) and 1 mg/mL Paneticin G418 (PAN-Biotech). Cells were used within four passages for experiments. Cells were regularly subjected to *Mycoplasma* testing. For in vitro drug assays, D4M cells were treated with 1 µM or 10 µM BRAFi PLX4720 for 24 hours. Dimethylsulfoxid (DMSO) was used as a vehicle.

### In vivo tumor growth studies

Zbtb46^GFP/WT^, C57BL/6N and Batf3^−/−^ mice were injected s.c. into flank skin with 3×10^5^ D4M cells in phosphate-buffered saline (PBS) (Thermo Fisher Scientific). Male and female mice (6–12 weeks) were used throughout the experiments of the study. When tumors were palpable (6 days after tumor transplantation), tumor growth was measured three times per week using a digital caliper by measuring the shortest and the longest diameter of the tumor. The tumor size was calculated according to the formula: shortest diameter × longest diameter. When tumors reached a size between 30 and 35 mm^2^, animals were fed with either control chow or BRAFi-containing chow (417 mg PLX4720/kg, Plexxikon, Berkeley, California, USA). PLX4720 was formulated into a rodent diet by Research Diets (New Brunswick, New Jersey, USA). Control chow-treated mice were designated as “untreated” (analyzed on day 14 after tumor transplantation). Tumors were referred to as “BRAFi-sensitive” when they decreased in size on 6 days of BRAFi therapy (analyzed on day 14 after tumor transplantation). Another group of animals were kept on BRAFi treatment until tumors regrew due to BRAFi resistance development and were referred to as “BRAFi-resistant” (analyzed on days 28–32 after tumor transplantation). BRAFi-resistant tumors were analyzed when they reached a size of approximately 90 mm^2^ (comparable to tumor size of untreated animals on day 14). For in vivo OT-I and OT-II T-cell proliferation assay, C57BL/6N were injected s.c into flank skin with 3×10^5^ D4M-OVA cells in PBS. For B16-OVA experiments, Zbtb46^GFP/WT^ mice were injected s.c. into flank skin with 1.5×10^5^ B16-OVA cells in PBS as described earlier.^70^

### Preparation of single-cell suspensions from tumors and tumor-draining LN

Tumors and tumor-draining LN were dissected from mice, mechanically disrupted and digested with 250 µg/mL collagenase D (Roche, Basel, Switzerland) and 300 µg/mL DNase I (Roche) in Hank’s Salt Solution (w/o Mg2^+^, Ca2^+^, PAN-Biotech) supplemented with 2% FCS. Tumors were digested for 45 min at 37°C and tumor-draining LN were digested for 25 min at 37°C. Digestion was stopped with 5 mM EDTA (Lonza, Basel, Switzerland) and tissue pieces were pressed through a 100 µm cell strainer (Corning, New York, USA) with a 2 mL syringe plunger (BD Biosciences, Franklin Lakes, New Jersey, USA) to obtain single-cell suspensions for flow cytometry analysis. Tumor-draining LN single-cell suspensions for the assessment of T-cell responses were generated by passing the tissue through a 100 µm cell strainer without prior digestion.

### Flow cytometry analysis of transplantable mouse tumors and tumor-draining LN

Cells were stained for 3 min at RT with the fixable viability dye eFluor780 (Thermo Fisher Scientific) or for 15 min with the Zombie NIR Fixable Viability Kit (BioLegend, San Diego, California, USA) to exclude dead cells, followed by incubation for 15 min with anti-mouse CD16/CD32 mAb (clone: 2.4G2, TONBO Biosciences, San Diego, California, USA) to prevent non-specific FcR-mediated antibody staining. Single-cell suspensions were incubated with a mix of fluorescently labeled monoclonal antibodies for 30 min at 4°C. Surface staining for chemokine receptors CCR2 and CCR7 was performed for 30 min at 37°C. Following the surface staining, cells were washed twice and analyzed directly or used for intracellular staining. For this purpose, cells were fixed and permeabilized using the Cytofix/Cytoperm Kit (BD Biosciences) according to the manufacturer’s protocol. For intracellular FoxP3 staining, cells were fixed and permeabilized for 45 min using the FoxP3 staining buffer kit according to the manufacturers’ protocol (eBioscience, Carlsbad, California, USA). Flow cytometry sample acquisition was performed on a 4 L or 5 L Aurora Spectral Flow Cytometer (Cytek Bioscience, Amsterdam, Netherlands) and a CytoFLEX S (Beckman Coulter Life Sciences, Krefeld, Germany). Flow Cytometry data was analyzed using FlowJo V.9 and V.10 (BD Biosciences) and in a cloud-based OMIQ analysis platform (OMIQ, Boston, Massachusetts, USA). See the full antibody list in online supplemental table 1.

### High-dimensional flow cytometry analysis

For dimensionality reduction of tumor-infiltrating myeloid cells using UMAP and FlowSOM clustering analysis,^41^ data analysis pipelines using OMIQ were built. Briefly, data from tumor tissue was cleaned by removing cellular debris, doublets and dead cells and gated on viable CD45^+^ cells using FlowJo. Next, NK cells, NKT cells, T cells and B cells were analyzed separately, based on the gating strategy shown in online supplemental figure 1c. Files containing the myeloid cells of interest were exported from FlowJo and used for further analysis using OMIQ. Data was arcsinh transformed (cofactor 6000). All fluorescence parameters, excluding viability dye eFluor780, CD45 BUV805, lymphoid cell markers (NK1.1 BB630, CD3 PE-Cy5, CD19 BB660) and markers for phenotypical characterization of myeloid cells (CD40 BB700, CCR7 APC, PD-L1 BUV615, PD-L2 APC-R700) were projected onto a two-dimensional plot by UMAP (neighbors=15, minimum distance=0.4, learning rate=1, epochs=200). Data set was clustered with FlowSOM (distance metric=euclidean) and resulting clusters were overlaid over UMAP plots. Clustered heatmaps showing fluorescence intensity were generated in OMIQ analysis platform.

### Multiplex immunohistochemistry

For staining of tumor sections, 7-color mIHC was performed on a Ventana Discovery Ultra instrument (Ventana Medical Systems, Basel, Switzerland) employing tyramide signal amplification-based OPAL technology (Akoya Biosciences, Marlborough, Massachusetts, USA) as previously described in detail.^71^ In brief, formalin-fixed paraffin-embedded tissue slides were deparaffinized and rehydrated at 69°C for 3×8 min in EZ Prep solution (Ventana Medical Systems) and heat-mediated antigen retrieval was performed at 95°C for 32 min in Cell Conditioning Solution 1 (Ventana Medical Systems). In six cycles, primary antibodies (listed in online supplemental table 2) were applied, followed by incubation of horseradish peroxidase-coupled secondary antibodies (Ventana Medical Systems) and OPAL reagents (Akoya Biosciences) at 36°C for 12 min and 8 min, respectively. Finalizing each cycle, antibody denaturation was performed at 100°C for 24 min in Cell Conditioning Solution 2 (Ventana Medical Systems). Slides were counterstained with DAPI (Merck, Darmstadt, Germany) for 8 min at RT and mounted in Fluoromount-G medium (SouthernBiotech, Birmingham, Alabama, USA). Multispectral images at 200-fold magnification were acquired by the Vectra V.3.0 Automated Imaging System (Akoya Biosciences). Downstream image processing was done with the inForm software (Akoya Biosciences). Therefore, fluorescence spectra were unmixed based on a manually built OPAL fluorophore library and cells were quantified using a trained algorithm to discriminate tissue from non-tissue areas, segment cells based on DAPI staining and phenotype cells based on pattern and intensity of the respective fluorescence signal. Data was further processed and analyzed using RStudio and R V.4.3.0 (R Core Team, Vienna, Austria) with the addins phenoptr^72^ and phenoptrReports^73^ as well as the packages tidyverse,^74^ ggplot2,^75^ and rstatix.^76^ Representative images were exported as multichannel Tagged Image File Format (TIFF) files and processed by applying arithmetic point operations in ImageJ.^77^ For whole tumor images, multichannel TIFFs were stitched together using QuPath.^78^

### In vivo T-cell depletion assay

To deplete CD4^+^ and CD8^+^ T cells in vivo, mice were treated intraperitoneally with 250 µg of anti-CD4 (clone GK1.5), 250 µg of anti-CD8 (clone 2.43) or IgG2b antibody control (all from Bio X Cell, New Hampshire, USA). Depletion was initiated 2 days before tumor transplantation and repeated 6 days after tumor transplantation. Depletion was verified by flow cytometry analysis in blood.

### Adoptive cell transfer of OT-I and OT-II T cells

For adoptive cell transfer of OVA-specific OT-I and OT-II cells, CD8^+^ T cells were isolated from CD45.1^+^ OT-I T-cell receptor (TCR) transgenic mice^79^ and CD4^+^ T cells from CD45.1^+^ OT-II TCR transgenic mice.^80^ LN and spleen were harvested and passed through a 100 µm cell strainer (Corning) to obtain single cell suspensions. Red blood cells were lysed using ammonium chloride lysis buffer (1.68 mM NH_4_Cl (Roth, Karlsruhe, Germany), 1 mM KHCO_3_ (Roth), and 0.1 mM EDTA (Lonza)) for 3 min at RT. CD8^+^ T cells were isolated by using anti-mouse-CD8-α (Ly-2) MicroBeads (Miltenyi Biotec, Bergisch Gladbach, Germany) and CD4^+^ T cells by using anti-mouse-CD4 (L3T4) MicroBeads (Miltenyi Biotec) according to the manufacturers’ instructions. For in vivo T-cell proliferation assays, isolated CD8^+^ T cells and CD4^+^ T cells were labeled with 0.4 µM CellTrace Violet (Thermo Fisher Scientific) in PBS for 3 min at RT and injected intravenously (1×10^6^ cells/mouse) into CD45.2^+^ D4M-OVA bearing recipient mice. Tumor-draining LN of recipient mice were harvested and CD8^+^ T cell and CD4^+^ T-cell proliferation and activation was analyzed by flow cytometry, 3 days and 5 days after the adoptive cell transfer, respectively.

### In vitro T-cell restimulation

Tumor-draining LN were collected from mice adoptively transferred with OT-I or OT-II T cells and single-cell suspensions were prepared by passing through a 100 µm cell strainer. Then, the cells were seeded in U-bottom 96-well-plates at 2×10^5^ cell density in Roswell Park Memorial Institute (RPMI) medium (PAN-Biotech) supplemented with 10% FCS, 2 mM L-glutamine (PAN-Biotech), and 50 µg/mL Gentamycin (Gibco). T cells were restimulated with 1 µM OVA_257-264_ or OVA_323-339_ peptide (Genaxxon, Ulm, Germany) for 48 hours. Culture supernatants were collected and stored at −80°C. IFN-γ was quantified using the mouse IFN-γ ELISA Kit (Thermo Fisher Scientific) following the manufacturers’ instructions.

### Cytokine quantification in D4M tumor lysates

To determine the expression of inflammatory cytokines, tumor samples were homogenized and resuspended in the presence of a protein inhibitor cocktail (Roche). Protein lysates from D4M tumor tissues were analyzed for the presence of cytokines by Bio-Plex technology. The concentration of the cytokines of tumor lysates were analyzed using the 36-ProcartaPlex (MAN0016936, Thermo Fisher Scientific) on a Bio-Plex 200 System (Bio-Rad, Munich, Germany) and calculated as pg cytokine per g tumor tissue.

### Capillary-based immunoblotting

Cells were lysed in Radio-Immunoprecipitation Assay (RIPA) buffer (Cell Signaling Technology, Danvers, Massachusetts, USA) supplemented with 1× cOmplete proteinase inhibitor cocktail and 1× PhosSTOP phosphatase inhibitors (Roche). For supernatant collection and sampling, FCS-free culture medium was used. The 12–230 kDa separation module capillary cartridges and the anti-mouse detection module (Protein Simple, San Jose, California, USA) was used to detect proteins in cell lysates and culture supernatants. In the Jess system, all steps from protein separation, immunoprobing, and chemiluminescent detection are fully automated and were conducted according to the manufacturer’s instructions. The results were analyzed with the software Compass for SW (V.6.2.0) and visualized as lane views of the electropherograms. Used antibodies are listed in online supplemental table 3.

### RNA isolation from tumor tissue

For RNA-sequencing (RNA-seq) analysis, D4M tumor samples were weighed and based on their weight RNeasy Midi (Qiagen, Hilden, Germany) (20–250 mg) or RNeasy Maxi Kit (Qiagen) (250 mg—1 g) was used for RNA isolation. Frozen samples were disrupted and homogenized on ice in an appropriate volume of lysis buffer according to the manufacturers’ recommendations by using a Tissue Ruptor (Qiagen) with disposable probes (Qiagen). RNA was isolated according to the manufacturers’ protocol by adding an additional DNAse I treatment (Qiagen). RNA elution step was repeated twice in the recommended total volume of either 300 µL (Midi Kit) or 1.6 mL (Maxi Kit) RNAse-free water and concentrated with an Eppendorf Concentrator Plus (Merck, Darmstadt, Germany) by using the vacufuge mode before further measurements. RNA amount and quality control was assessed via NanoDrop (Thermo Fisher Scientific) and Qubit (Thermo Fisher Scientific) measurements. RNA integrity was validated via Bioanalyzer (Agilent, Santa Clara, California, USA) before library preparations.

### RNA sequencing analysis

Lexogen QuantSeq 3’-messenger RNA libraries were prepared at the MultiOmics Sequencing Core Facility (Medical University of Innsbruck) following the manufacturer’s instructions (Lexogen GmbH, Vienna, Austria), quality validated, multiplexed and sequenced with an Illumina NovaSeq sequencer (Azenta, Leipzig, Germany) at 150 bp read length.

### RNA-seq data processing and visualization

RNA-seq data was processed with the nf-core RNA-seq pipeline V.3.10.1 (https://nf-co.re/rnaseq/3.10.1) using default parameters. Processing encompassed data quality control, read alignment, and gene expression quantification. Subsequently, raw gene counts were imported into R V.4.2.3 for differential gene expression analysis using the DESeq2 package (V.1.38.3).^81^ Heatmaps were generated using ggplot2 (V.3.4.2).^75^ Analysis and visualization of Gene Ontology (GO) terms associated with differentially-expressed genes was performed using Metascape.^82^ Both groups of genes (upregulated and downregulated, adjusted p value<0.05) by a Log2FC>1 were used for GO-derived biological processes, molecular functions and cellular components. The biological terms are grouped together based on their shared genes where the similarity between terms is calculated using kappa statistics.

### Statistical analysis

Statistical analysis was performed using GraphPad Prism V.9.0 (GraphPad Software, San Diego, California, USA). To determine whether parametric or non-parametric statistical tests are appropriate, data sets were examined for normality using D’Agostino-Pearson test. For more groups, statistical significance was determined with one‐way analysis of variance followed by Tukey’s multiple comparison test (parametric) or Kruskal-Wallis test followed by Dunn’s multiple comparison test (non-parametric). A p value of <0.05 was considered statistically significant (*), <0.01 very significant (**), <0.001 highly significant (***) and <0.0001 extremely significant (****). Data is presented as mean±SEM.

## Data Availability

All data relevant to the study are included in the article or uploaded as supplementary information. The bulk RNAseq data sets will be made available upon acceptance of paper.
