# Supplementary material for: Tumor-targeted therapy with BRAF-inhibitor recruits activated dendritic cells to promote tumor immunity in melanoma
Source: J Immunother Cancer. 2024 Apr 17;12(4):e008606. doi: 10.1136/jitc-2023-008606 (PMC11029477; doi:10.1136/jitc-2023-008606)
Supplement: Supplementary data [file jitc-2023-008606supp001.pdf]

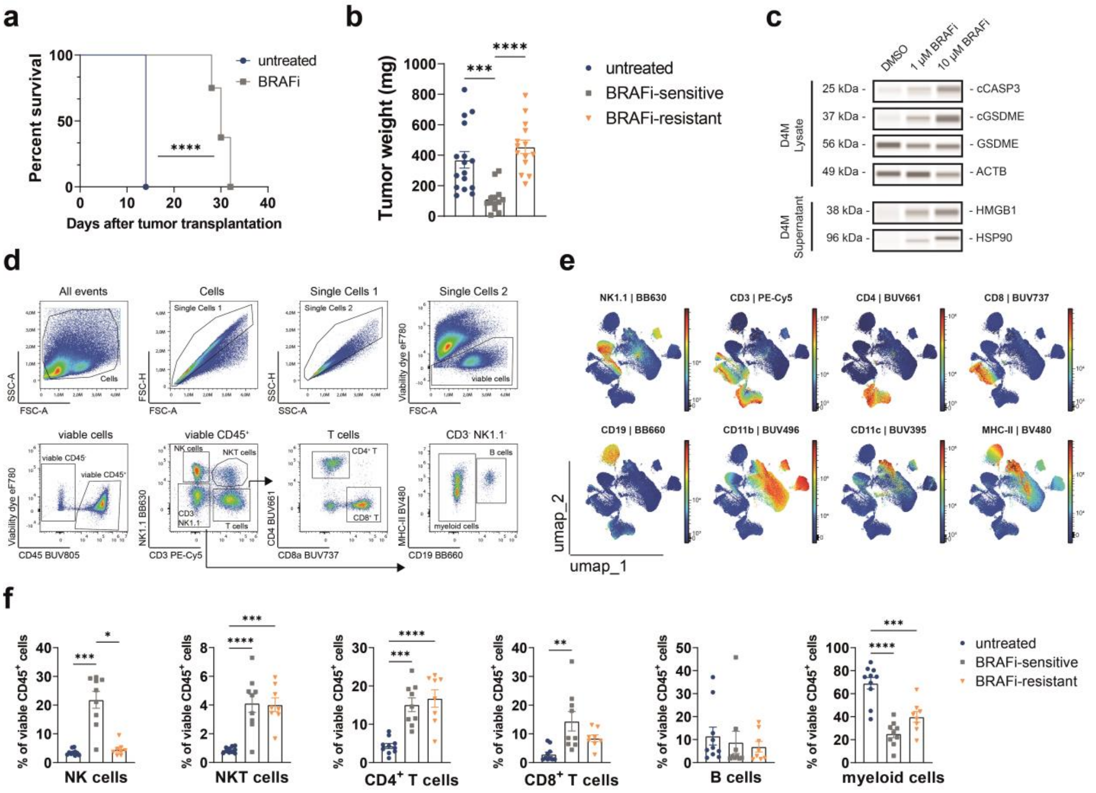

Supplementary Figure 1. BRAFi treatment mediates strong immunomodulatory activity. 3 x 10^5 D4M melanoma cells were s.c. injected into the flank skin of Zbtb46$^{GFP/WT}$ mice. When tumors reached a size of 30-35 mm at day 8 after transplantation, mice received either BRAFi-containing or control chow. On day 14, untreated and BRAFi-sensitive D4M tumors were analyzed. Another group of mice kept on the BRAFi-containing diet started to regrow tumors due to BRAFi-resistance (analyzed on day 28-32 after transplantation when tumors were comparable in size to untreated ones, BRAFi-resistant). **a** Kaplan-Meier survival curve of 3 independent experiments is shown (n ≥ 9/group). **b** Tumor weight was measured at the time point of analysis (3 independent experiments, n ≥ 13/group). **c** D4M cells were treated with 1 µM or 10 µM BRAFi PLX4720 *in vitro* for 24 hours. Shown are levels of cleaved caspase 3 and GSDME (full-length GSDME and cleaved GSDME) in cell lysates and levels of HMGB1 and HSP90 in supernatant from D4M cells. A representative lane view of the electropherograms of two independent experiments is shown. DMSO was used as vehicle. **d** Gating strategy for flow cytometry analysis of D4M tumor tissue. After excluding cellular debris, doublets and dead cells, viable CD45$^+$ cells were gated. CD3$^+$ T cells, NK1.1$^+$ NK cells and CD3$^+$ NK1.1$^+$ NKT cells were identified. Inclusion of CD4 and CD8 allowed further characterization of T cell subsets. B cells were characterized by their expression of CD19 and MHC-II. The remaining CD19$^-$ cells were considered as myeloid cells. Shown is a gating tree using a representative BRAFi-sensitive tumor. CD4$^+$ T: CD4$^+$ T cells, CD8$^+$ T: CD8$^+$ T cells. **e** Flow cytometry data from untreated, BRAFi-sensitive and – resistant tumors derived from Zbtb46$^{GFP/WT}$ mice was concatenated. Shown is a UMAP dimensionality reduction of all cells and groups displaying the surface expression of NK1.1, CD3, CD4, CD8, CD19, CD11b, CD11c, and MHC-II. **f** Flow cytometry analysis of tumor-infiltrating NK cells, NKT cells, CD4$^+$ T cells, CD8$^+$ T cells, B cells and myeloid cells from untreated, BRAFi-sensitive and -resistant tumors. Percentages are shown as summary graphs from three independent experiments (n ≥ 8/group). Statistical significance was determined using one-way ANOVA followed by Tukey's multiple comparison test or Kruskal-Wallis test followed by Dunn's multiple comparison test. Graphs show the mean ± SEM. *p<0.05; **p<0.01; ***p<0.001; ****p<0.0001;

a

	CD3	CD8	CD4	GrzB	PD-1	FoxP3	Parent
CD3⁺ T cells	+	+|-	+|-	+|-	+|-	+|-	
CD8⁺ T cells	+	+	-	+|-	+|-	+|-	CD3⁺ T cells
CD4⁺ T cells	+	-	+	+|-	+|-	+|-	CD3⁺ T cells
GrzB⁺ CD8⁺ T cells	+	+	-	+	+|-	-	CD8⁺ T cells
PD-1⁺ CD8⁺ T cells	+	+	-	+|-	+	-	CD8⁺ T cells
GrzB⁺ CD4⁺ T cells	+	-	+	+	+|-	-	CD4⁺ T cells
PD-1⁺ CD4⁺ T cells	+	-	+	+|-	+	-	CD4⁺ T cells
FoxP3⁺ CD4⁺ T cells	+	-	+	+|-	+|-	+	CD4⁺ T cells

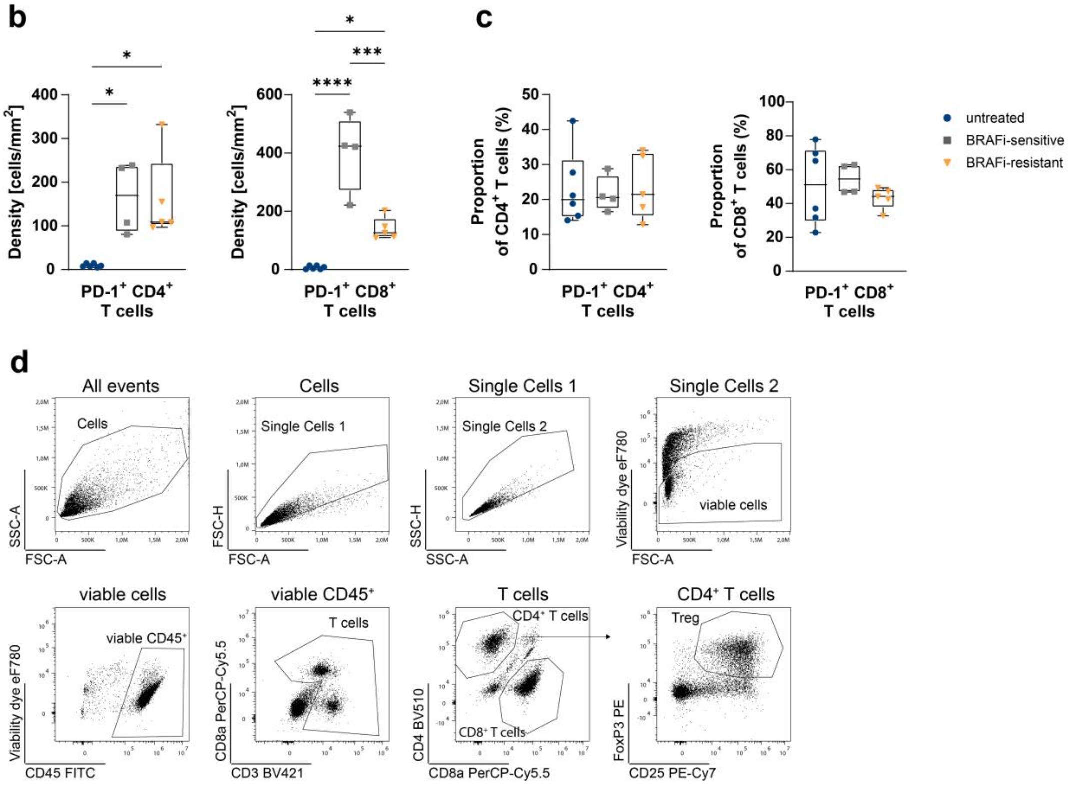

Supplementary Figure 2. Characterization of tumor-infiltrating T cells. a Overview of all markers included in the mIHC panel defining the different T cell phenotypes. **b** Densities [cells/mm^2] of CD4$^+$ and CD8$^+$ T cells positive for PD-1. **c** Proportions of CD4$^+$ and CD8$^+$ T cells positive for PD-1. **d** Gating strategy to identify regulatory T cells in D4M tumor cell suspensions by flow cytometry. Immune cells are pre-gated on single, viable cells, then CD45 for all immune cells, followed by T cells (CD3$^+$). T cells were subdivided into CD8$^+$ and CD4$^+$ T cells and subsequently Tregs were identified by their expression of CD25 and FoxP3. For (**b,c**) results from ≥4 mice/group are shown. Statistical significance was determined using one-way ANOVA followed by Tukey's multiple comparison test or Kruskal-Wallis test followed by Dunn's multiple comparison test. Graphs show the mean ± SEM. *p<0.05; ***p<0.001; ****p<0.0001;

Hornsteiner F, *et al. J Immunother Cancer* 2024; 12:e008606. doi: 10.1136/jitc-2023-008606

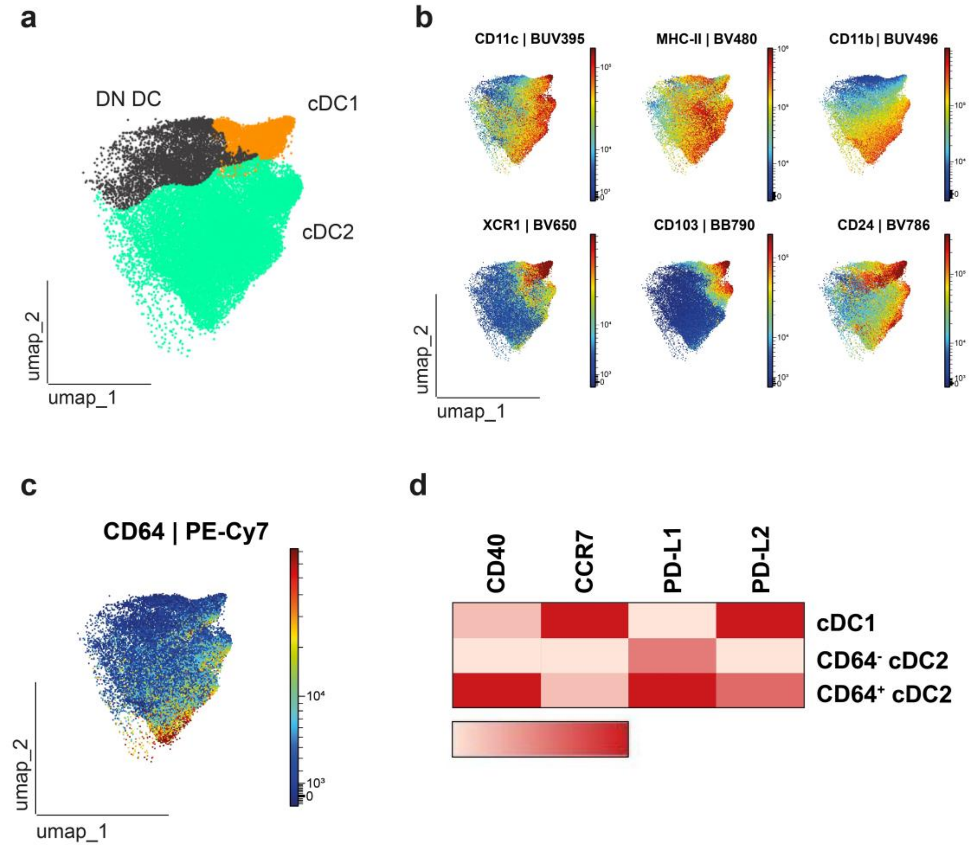

Supplementary Figure 3. Flow cytometry data of D4M tumors derived from Zbtb46$^{GFP/WT}$ mice. a-c Data from untreated, BRAFi-sensitive and – resistant tumors was concatenated. UMAPs of all cells and all groups showing the three DC clusters. **a** UMAP plot showing cDC1, cDC2 and DN DC from FlowSOM clustering described in Fig. 3. **b** UMAP showing the surface expression of CD11c, MHC-II, CD11b, XCR1, CD103 and CD24. **c** UMAP dimensionality reduction displaying the expression of CD64. **d** Heatmap displaying the expression of CD40, CCR7, PD-L1 and PD-L2 on tumor-infiltrating DC populations as MFI across all three different tumor stages. Results from three independent experiments are shown (n ≥ 8 mice/group).

Hornsteiner F, *et al. J Immunother Cancer* 2024; 12:e008606. doi: 10.1136/jitc-2023-008606

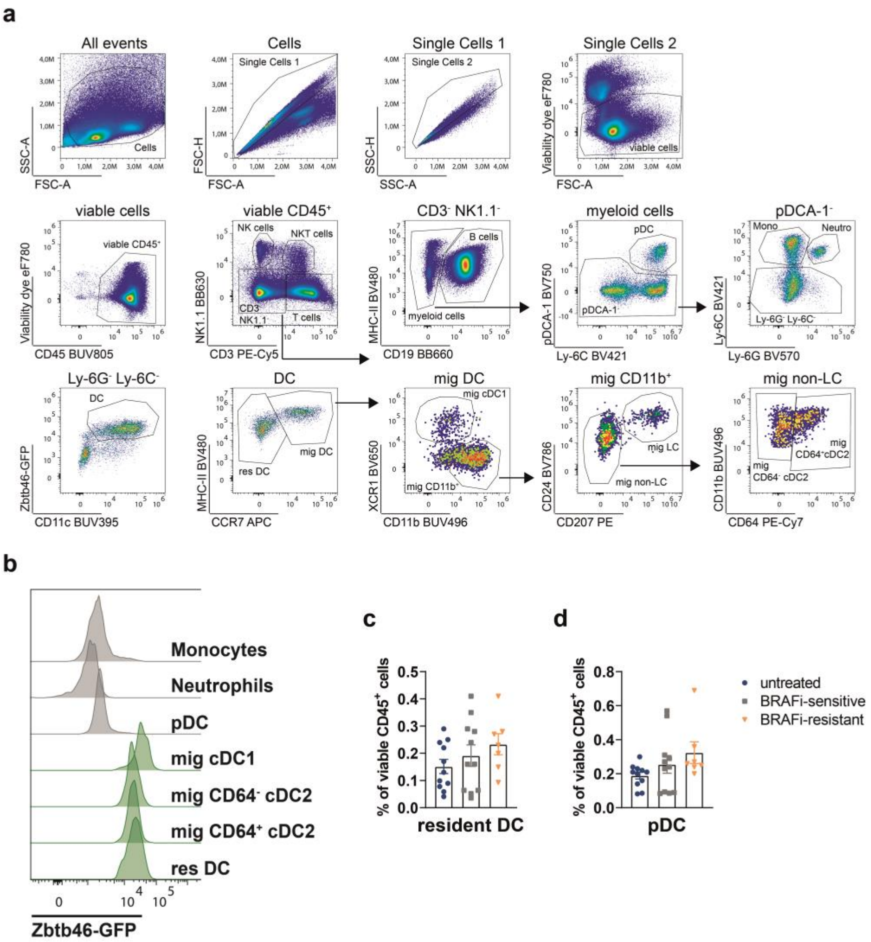

Supplementary Figure 4. Characterization of DC in tumor-draining LN. a Gating strategy for flow cytometry analysis of tumor-draining LN. After exclusion of of cellular debris, doublets, and dead cells, viable CD45$^+$ cells were gated. CD3$^+$ T cells, NK1.1$^+$ NK cells, CD3$^+$ NK1.1$^+$ NKT cells and CD19$^+$ MHC-II$^+$ B cells were excluded. From the remaining myeloid cells, pDC were characterized (pDCA-1$^+$ Ly-6C$^+$). Next, monocytes (Ly-6C$^+$ Ly-6G$^-$) and neutrophils (Ly-6C$^+$ Ly-6G$^+$) were discriminated and the remaining cells were gated with CD11c versus Zbtb46-GFP to identify DC. DC were subdivided into migratory DC (MHC-II^{high} CCR7$^+$) and resident DC (MHC-II$^+$ CCR7$^-$). Migratory DC consist of cDC1 (XCR1$^+$ CD11b$^-$) and CD11b$^+$ DC. CD11b expressing cells contain LC (CD24$^+$ CD207$^+$). The remaining CCR7$^+$ cells (CD24$^-$ CD207$^-$) can be separated into migratory CD64$^-$ cDC2 and migratory CD64$^+$ cDC2, Mono: monocytes, Neutro: neutrophils, mig DC: migratory DC, res DC: resident DC, LC: Langerhans cells. **b** Representative single-color histogram for Zbtb46-GFP expression of the different gated myeloid cell populations to verify correct gating of DC. **c** Percentages of resident DC in tumor-draining LN from untreated, BRAFi-sensitive and -resistant tumors. **d** Percentages of pDC in tumor-draining LN from untreated, BRAFi-sensitive and -resistant tumors. For (**c,d**), results from from three independent experiments are shown (n ≥ 7 mice/group). Statistical significance was determined using one-way ANOVA followed by Tukey's multiple comparison test or Kruskal-Wallis test followed by Dunn's multiple comparison test. Graphs show the mean ± SEM.

Hornsteiner F, *et al. J Immunother Cancer* 2024; 12:e008606. doi: 10.1136/jitc-2023-008606

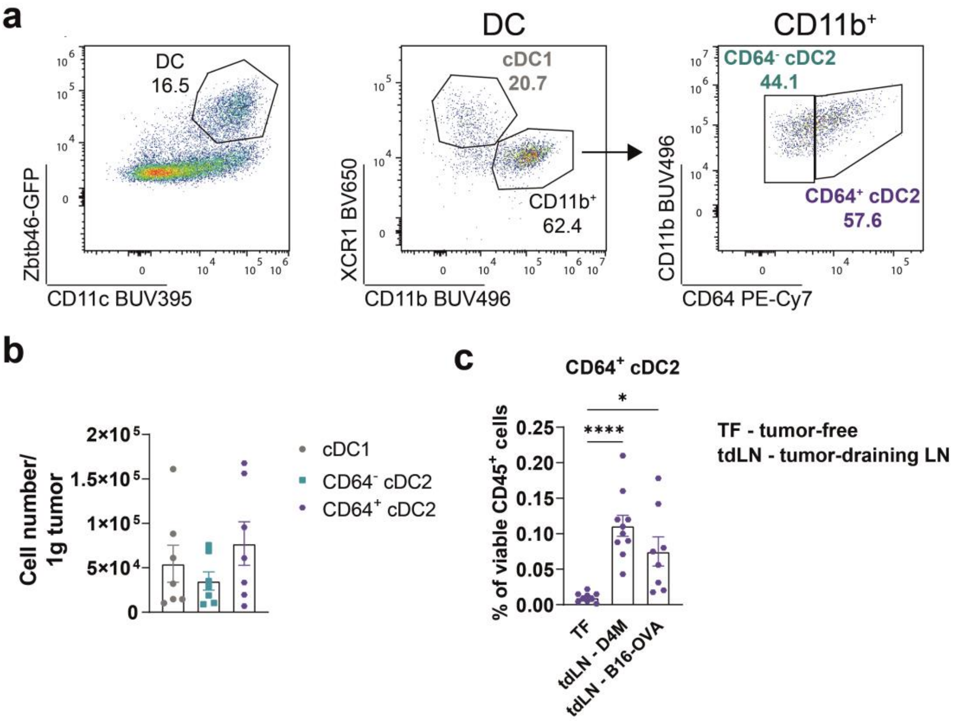

Supplementary Figure 5. CD64$^+$ cDC2 are induced in melanoma. 3 x 10^5 D4M melanoma cells or 1.5 x 10^5 B16-OVA melanoma cells were s.c. injected into the flank skin of Zbtb46$^{GFP/WT}$ mice. When tumors reached a size of 80-100 mm^2 tumors and tumor-draining LN were analyzed. **a** Manual gating of DC subsets in B16-OVA tumors. Pregate on CD45$^+$ lineage-negative (CD3$^-$ NK1.1$^-$ CD19$^-$), followed by gating on Ly-6C$^-$ Ly-6G$^-$ MerTK$^-$ F4/80^{low} cells to exclude monocytes, neutrophils and TAM. DC were identified by gating CD11c versus Zbtb46-GFP. Tumor-infiltrating DC consist of cDC1 (XCR1$^+$ CD11b$^-$) and cDC2 (XCR1$^-$ CD11b$^+$). From the CD11b$^+$ subset, CD64$^+$ cDC2 were identified. **b** Cell numbers of tumor-infiltrating cDC1, CD64$^-$ cDC2 and CD64$^+$ cDC2 in B16-OVA are shown. **c** DC subsets were gated accordingly to Fig. 5a. Percentages of CD64$^+$ cDC2 in skin-draining LN of tumor-free Zbtb46$^{GFP/WT}$ mice (TF) and tumor-draining LN (tdLN) of untreated D4M and B16-OVA bearing Zbtb46$^{GFP/WT}$ mice are shown. Results from at least two independent experiments are shown (n ≥ 7 mice/group). Statistical significance was determined using one-way ANOVA followed by Tukey's multiple comparison test or Kruskal-Wallis test followed by Dunn's multiple comparison test. Graphs show the mean ± SEM. *p<0.05; ****p<0.0001;

Hornsteiner F, *et al. J Immunother Cancer* 2024; 12:e008606. doi: 10.1136/jitc-2023-008606

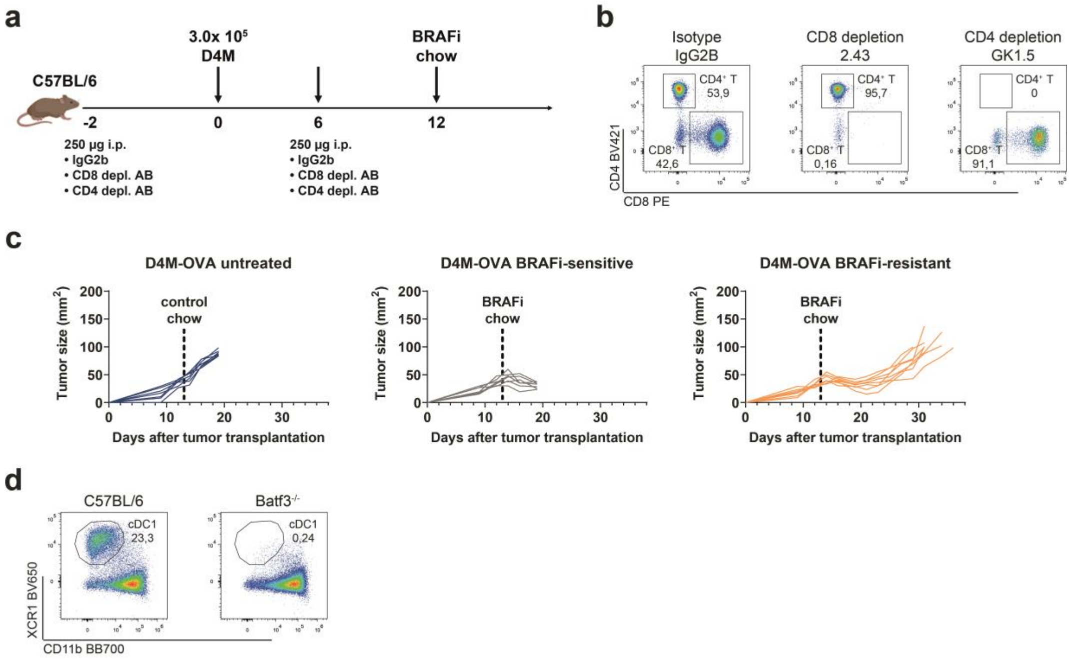

Supplementary Figure 6. T cells contribute to maintaining tumor regression by BRAFi. a Experimental design of the T cell depletion assay. Depletions were initiated 2 days before tumor transplantation and were repeated 6 days after tumor transplantation. **b** Verification of T cell depletion in blood before transplantation of tumors. $CD4^+$ T: $CD4^+$ T cells, $CD8^+$ T: $CD8^+$ T cells. **c** 3 x 10^5 D4M-OVA melanoma cells were s.c. injected into the flank skin of $Zbtb46^{GFP/WT}$ mice. When tumors reached a size of 30-35 mm^2 on day 13 after transplantation, mice received either BRAFi-containing or control chow. The group of mice kept on the BRAFi-containing diet started to regrow tumors due to BRAFi-resistance. Individual D4M-OVA tumor growth is shown (n ≥ 7/group). **D** Flow cytometry analysis of spleen to verify the lack of cDC1 in $Batf3^{-/-}$ mice.

Hornsteiner F, *et al. J Immunother Cancer* 2024; 12:e008606. doi: 10.1136/jitc-2023-008606

Supplementary Table 1. Antibodies used for flow cytometry

Antibody	Clone	Manufacturer	Identifier
NK1.1 BB630	PK136	BD	Custom-made
CD19 BB660	1D3	BD	Custom-made
CD40 BB700	3/23	BD	742136
CD103 BB790	M290	BD	Custom-made
CD207 PE	929F3.01	self-conjugated	
F4/80 PE-Dazzle594	BM8	Biolegend	123146
CD3 FITC	17A2	BD	555274
CD3e PE-Cy5	145-2C11	BD	553065
CD3 BV421	145-2C11	Biolegend	100227
CD3 APC	17A2	Biolegend	100236
CD64 PE-Cy7	X54	Biolegend	139314
CCR7 APC	4B12	Biolegend	120108
PD-L2 APC-R700	TY25	BD	Custom-made
PD-L2 R718	TY25	BD	752227
Ly-6C BV421	HK1.4	Biolegend	128031
MHC class II BV480	M5/114	BD	566086
Ly-6G BV570	1A8	BD	Custom-made
XCR1 BV650	ZET	Biolegend	148220
CCR2 BV711	475301	BD	747964
pDCA-1 BV750	927	BD	747608
CD24 BV786	M1/69	BD	744470
CD11c BUV395	N418	BD	744180
CD11b BUV496	M1/70	BD	749864
CD11b BB700	M1/70	BD	566416
MerTK BUV563	108928	BD	Custom-made
PD-L1 BUV615	MIH5	BD	752339
CD4 BUV661	RM4-5	BD	741461
CD4 BV421	GK1.5	Biolegend	100433
CD4 BV510	RM4-5	Biolegend	100553
CD8a BUV737	53-6.7	BD	612759
CD8a PE	53-6.7	Biolegend	100708
CD8a PerCP-Cy5.5	53-6.7	Biolegend	100734
CD45 BUV805	30-F11	BD	748370
CD45 FITC	30-F11	Biolegend	103108
CD25 PE-Cy7	PC61	Biolegend	102015
FoxP3 PE	FJK-16s	eBioscience	12-5773-82
CD45.1 FITC	A20	BD	553775
Vbeta 5.1, 5.2 PerCP-Cy5.5	MR9-4	Biolegend	139509
CD44 BV605	IM7	Biolegend	103047

Hornsteiner F, *et al. J Immunother Cancer* 2024; 12:e008606. doi: 10.1136/jitc-2023-008606

Supplementary Table 2. Primary antibodies and OPAL fluorophores used for mIHC

Antibody	Clone	Manufacturer	Incubation primary antibody	Fluorophore Dilution
PD-1 OPAL 650	D7D5W	Cell Signaling Technology	RT, 16 min	1:100
CD8 OPAL 620	D4W2Z	Cell Signaling Technology	RT, 32 min	1:100
CD4 OPAL 570	EPR19514	Abcam	RT, 32 min	1:700
CD3 OPAL 520	E4T1B	Cell Signaling Technology	RT, 32 min	1:100
Granzyme B OPAL 540	E5V2L	Cell Signaling Technology	RT, 32 min	1:350
FoxP3 OPAL 690	D6O8R	Cell Signaling Technology	36 °C, 32 min	1:100

Supplementary Table 3. Antibodies used for capillary-based immunoblotting

Antibody	Clone	Specificity	Manufacturer	Identifier
anti-β-actin (ACTB)	Polyclonal	Rabbit	Abcam	ab8227
anti-heat shock protein 90 α/β (HSP90)	F-8	Rabbit	Santa Cruz Biotechnology	sc-13119
anti-high mobility group box 1 (HMGB1)	polyclonal	Rabbit	Novus Biologicals	NB100-2322
anti-cleaved caspase 3 (cCASP3)	5A1E	Rabbit	Cell Signaling Technology	9664
anti-gasdermin E (GSDME)	EPR19859	Rabbit	Abcam	ab215191

Hornsteiner F, *et al. J Immunother Cancer* 2024; 12:e008606. doi: 10.1136/jitc-2023-008606
